# MED26 facilitates Mediator-Pol II complex assembly and promotes nuclear stress body formation during heat stress

**DOI:** 10.1016/j.isci.2026.117557

**Published:** 2026-09-23

**Authors:** Kexin Li, Kazuki Furugori, Hidefumi Suzuki, Ryota Abe, Shun Sawatsubashi, Nanami Yasui, Yoko Ike, Keiko Horiuchi, Hidehisa Takahashi

**Affiliations:** 1 Department of Molecular Biology, Yokohama City University Graduate School of Medicine, 3-9 Fukuura, Kanazawa-ku, Yokohama, Kanagawa 236-0004, Japan; 2 Department of Neurology and Stroke Medicine, Yokohama City University Graduate School of Medicine, 3-9 Fukuura, Kanazawa-ku, Yokohama 236-0004, Japan; 3 Research and Innovation Liaison Office, Institute for Advanced Medical Sciences, Tokushima University, 3-18-15 Kuramoto-cho, Tokushima, Tokushima 770-8503, Japan; 4 Translation Animal Model Innovation Core, Institute for Emerging Medical Innovation, Advanced Research Fields, Okayama University, 2-5-1 Shikata-cho, Kita-ku, Okayama 700-8558, Japan

**Keywords:** Mediator, MED26, MED15, HSF1, nuclear stress bodies

## Abstract

Cells adapt to environmental stress by reprogramming gene expression. During heat stress, the transcription factor heat shock factor 1 (HSF1) translocates into the nucleus and activates heat shock-responsive loci, including the long non-coding RNA HSATIII, promoting nuclear stress body (nSB) formation. We show that the Mediator complex subunit MED26 enhances HSATIII transcription and nSB formation through a mechanism distinct from its role at heat-shock response (HSR) genes. Under heat stress, MED26-containing Mediator is recruited to HSR genes and nSBs through interactions with HSF1 and MED15. Phosphorylation of the intrinsically disordered region of HSF1 appears to facilitate Mediator recruitment to nSBs. Acute loss of MED26 leads to the dissociation of the Mediator and RNA polymerase II (Pol II) from HSR gene promoters, whereas the Mediator remains bound to HSATIII despite Pol II dissociation. These results suggest that MED26 acts as molecular switch, thereby facilitating Mediator-Pol II complex assembly at nSBs and promoting nSB formation during heat stress.

## Highlights

• MED26 relocates from Cajal bodies to nuclear stress bodies upon heat stress
• HSF1 recruits MED26-containing Mediator through MED15 during heat stress
• Mediator recruitment differs between HSATIII and HSR genes
• MED26 facilitates Mediator-Pol II assembly to drive nuclear stress body formation

## Graphical abstract

**Figure:**
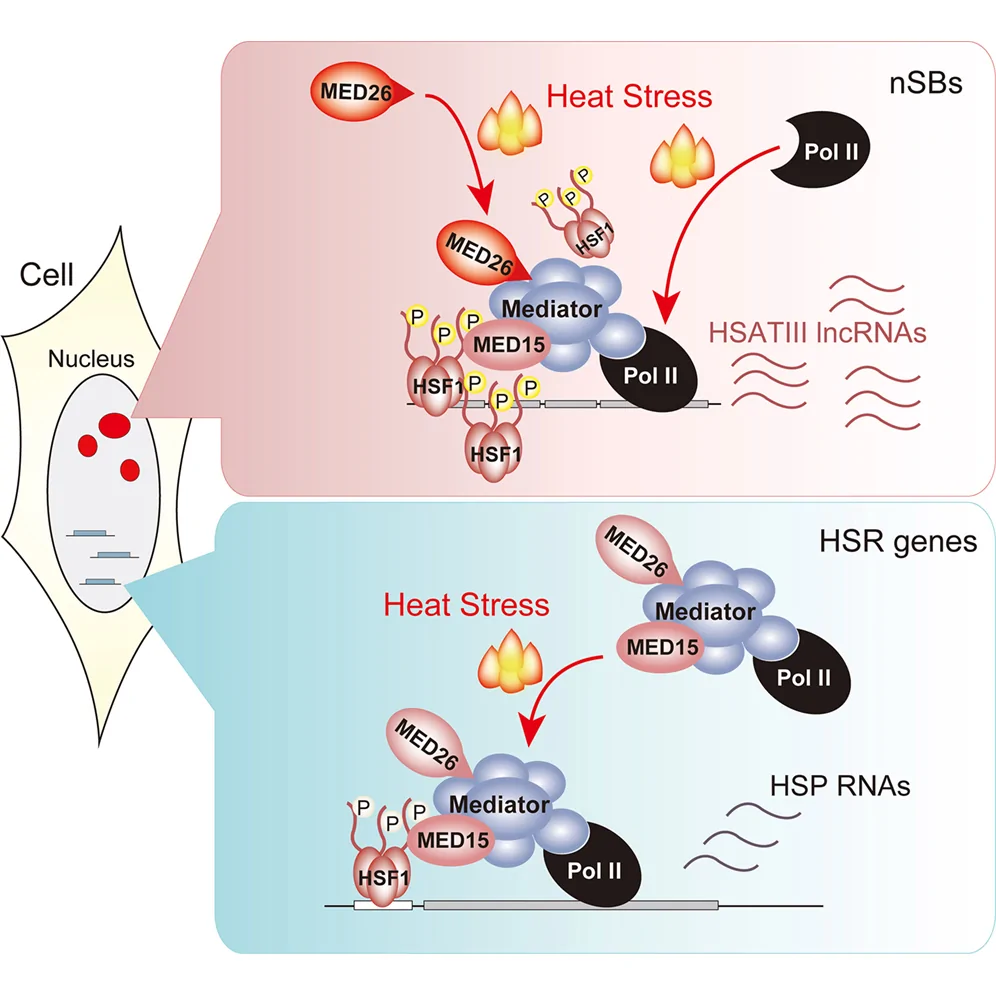

## Teaser

Biological sciences; Molecular biology; Cell biology

## Introduction

Cells respond to diverse stresses by dynamically modulating gene expression. As one of the most highly conserved stress pathways, the heat-shock response (HSR) is activated by elevated temperatures and other stimuli, including hypoxia, heavy metal exposure, and various pathological conditions.^1^ Elucidating HSR mechanisms is crucial because the chaperone proteins induced by heat shock are essential for proteostasis,^2^ and heat shock protein (HSP)70 has been implicated in neuron-glia communication within the *Caenorhabditis elegans* nervous system.^3^

Heat shock factors (HSFs) constitute a conserved family of DNA-binding transcription factors encoded by multiple human genes, including *HSF1*, *HSF2*, *HSF4*, *HSF5*, *HSFX*, and *HSFY*, which display distinct developmental, tissue-specific, or sex-specific expression patterns across species.^4^^,^^5^^,^^6^^,^^7^^,^^8^ HSF1, which is cytoplasmic and HSP-bound under basal conditions, is the master regulator of the HSR, while other HSFs function in more specialized biological contexts.^5^^,^^6^^,^^7^^,^^8^ Structurally, HSF1 contains a DNA-binding domain (DBD), oligomerization domains containing heptad repeats (HRs; HR-A/B) that mediate trimer formation, and a transcriptional activation domain (TAD), together with an intrinsically disordered regulatory domain (RD) whose post-translational modifications during heat stress are critical for HSF1 activation and condensate formation.^9^^,^^10^^,^^11^ The HR-C domain within the leucine zipper 4 region antagonizes oligomerization under non-stress conditions, thereby maintaining HSF1 in an inactive state.^12^

Upon exposure to heat stress, HSF1 dissociates from its chaperone regulators—HSP40, HSP70, and HSP90—trimerizes, translocates into the nucleus, and binds heat shock elements (HSEs) at target loci.^4^^,^^13^ HSF1 then activates two major RNA polymerase II (Pol II)-dependent transcriptional programs. In the canonical pathway, HSF1 induces HSR gene transcription by forming complexes with minimal transcriptional activity and recruiting transcriptional cofactors, including the Mediator complex.^10^ In parallel, heat stress induces robust Pol II-dependent transcription of the primate-specific long non-coding RNA (lncRNA) HSATIII located in pericentric regions, which, together with HSF1, nucleates the formation of membraneless nuclear stress bodies (nSBs).^14^^,^^15^^,^^16^

nSBs contribute to stress-induced gene expression reprogramming by sequestering factors involved in transcription and RNA processing, particularly splicing regulators.^17^^,^^18^ Recent studies have revealed that nSBs also function as reaction platforms that promote phosphorylation of Ser/Arg-rich splicing factors (SRSFs) during heat stress recovery, a process partly regulated by temperature-dependent activation of the kinase CLK1.^19^^,^^20^ Additionally, HSATIII transcripts are highly modified by N^6^-methyladenosine (m^6^A) through recruitment of the m^6^A writer complex to nSBs, resulting in sequestration of the nuclear m^6^A reader YTHDC1 and global repression of splicing during recovery.^18^^,^^20^ Despite extensive characterization of nSB functions during heat stress recovery, the transcriptional regulatory mechanisms governing the initial formation of nSBs remain poorly understood.

Beyond classical molecular interactions, recent studies have demonstrated that transcriptional regulation is governed by assemblies formed through liquid-liquid phase separation (LLPS) of transcription factors and coactivators containing intrinsically disordered regions (IDRs).^21^^,^^22^^,^^23^ The Mediator complex has been implicated in LLPS-dependent super-enhancer formation^24^^,^^25^ and heat stress-induced genome reorganization through dynamic cluster formation with Hsf1 and Pol II in yeast.^26^ Mediator, an evolutionarily conserved coactivator complex with 31 subunits in mammalian cells, is organized into head, middle, tail, and CDK8 kinase modules.^27^^,^^28^^,^^29^ Mediator exists as multiple functionally distinct forms, depending on the presence or absence of specific subunits. Among subunits, MED26 is incorporated into a sub-population of Mediator complexes that associate with RNA Pol II at near-stoichiometric levels.^30^^,^^31^^,^^32^ Although MED26 is correlated with the Mediator-Pol II association, it is not essential for the conversion of a Mediator into an active form.^33^ Furthermore, MED26- and kinase module-containing Mediators are mutually exclusive; mechanistically, the incorporation of MED26 into Mediator facilitates dissociation of the kinase module, promoting the formation of a Pol II-associated Mediator complex.^27^^,^^34^ MED26 also facilitates Pol II transcription elongation by recruiting the little elongation complex (LEC) and super elongation complex (SEC) to distinct genes.^35^^,^^36^^,^^37^ These findings raise the possibility that MED26 functions in a context-dependent manner.

HSR genes and HSATIII lncRNA genes are transcribed by Pol II during the HSR, and functional interactions between HSF1 and Mediator have been reported across species. In yeast, tail-module subunits Med15 and Med16 interact with Hsf1 at *HSP* promoters during heat stress.^38^ In *Drosophila*, the head-module subunit Med17 (TRAP80) interacts with Hsf,^39^^,^^40^ while in plants, differential recruitment of MED14/17 is critical for effective HSRs.^41^ In human cells, MED12 was recently shown to promote HSF1 condensate formation on HSE arrays.^42^^,^^43^ These findings indicate that the Mediator is involved in the transcriptional regulation of HSR genes during heat stress; however, its roles in the transcriptional control of HSATIII lncRNA genes and nSB formation remain unclear.

Here, we identify Mediator subunit MED26 as a critical promoter of HSATIII transcription and nSB formation through a mechanism distinct from its role at HSR genes. Upon heat stress, MED26-containing Mediator (MED26-Mediator) is recruited to both HSR gene loci and HSATIII-associated nSBs via interactions with HSF1 and MED15, and this recruitment is enhanced by phosphorylation of the HSF1 IDR. Acute MED26 depletion results in the loss of both Mediator and Pol II from HSR gene promoters, whereas the Mediator is retained at HSATIII loci despite Pol II dissociation. Thus, MED26 appears to function as a ligand-like molecular switch, stabilizing Mediator-Pol II complex formation at nSBs to promote nSB formation during heat stress.

## Results

### Heat stress triggers re-localization of MED26-Mediator from Cajal bodies to nSBs

MED26-Mediator colocalizes with LEC at Cajal bodies (CBs) and promotes the transcription of genes encoding polyA-less mRNAs, such as small nuclear RNAs.^36^ Additionally, MED26 interacts with SEC through its N-terminal domain (NTD) to facilitate the transcription elongation of genes encoding polyA-plus mRNAs.^35^^,^^37^ Because MED26 contributes to the Mediator-mediated recruitment of Pol II and transcription elongation of diverse genes,^27^^,^^35^^,^^36^ we focused on its role in the HSR in HeLa cells. Immunofluorescence (IF) staining showed that heat stress resulted in weaker MED26 signals colocalizing with CBs and the redistribution of MED26 into larger nuclear foci. In contrast, CBs lacking detectable MED26 became enlarged upon heat stress (Figures 1A and 1B). Next, to examine heat stress-induced changes in MED26-interacting proteins, we performed immunoprecipitation followed by mass spectrometry (IP-MS) using a cell line stably expressing FLAG-tagged MED26. Consistent with the IF results, MED26 dissociated from components of the LEC upon heat stress (Figure 1C). Previous studies have shown that, upon heat shock, RNA-binding proteins known to associate with HSATIII lncRNA, including scaffold attachment factor B (SAFB), SRSFs, and heterogeneous nuclear ribonucleoproteins (HNRNPs), are recruited to nSBs.^16^^,^^18^^,^^19^ We found that the interaction between MED26 and these nSB-associated proteins increased significantly during heat stress (Figure 1C), suggesting that the heat shock-induced MED26-containing nuclear foci correspond to nSBs. In addition, MED26 is known to colocalize with the LEC in CBs together with other Mediator subunits.^44^ Proteomic analysis using the stable FLAG-MED26-expressing cell line revealed that the association between MED26 and other Mediator subunits did not differ substantially between untreated and heat-stress conditions (Figure S1A). Taken together, these results indicate that MED26-containing Mediator is relocalized from CBs to nSBs upon heat stress.

**Figure 1:**
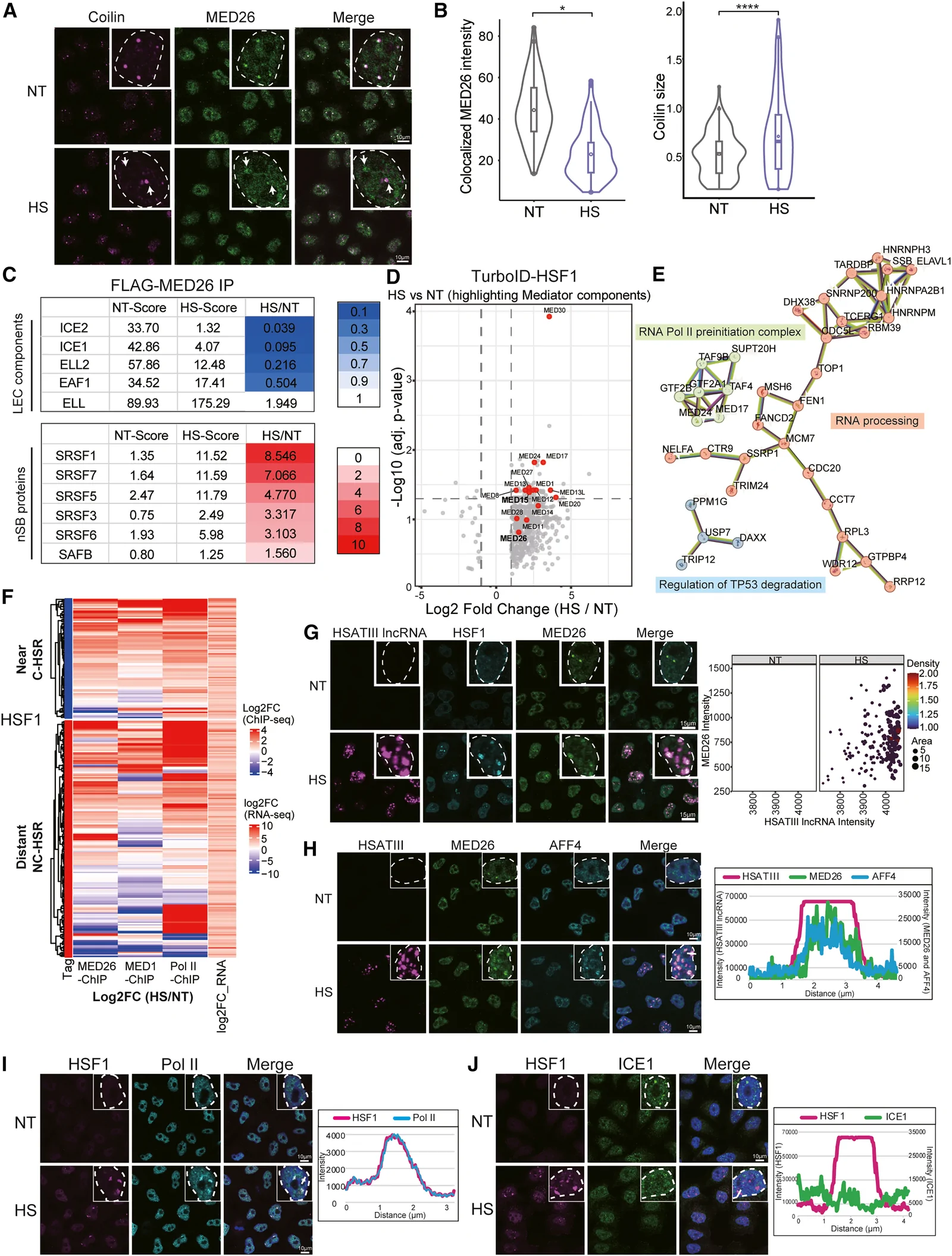
Heat stress triggers re-localization of MED26-containing Mediator from transcriptionally active nuclear bodies to nSBs by interacting with HSF1 (A) Fluorescent immunostaining images of Coilin (magenta) and MED26 (green), showing their localization under normal temperatures (NTs) and heat shock (HS). Dashed lines indicate the nucleus, and arrows indicate the larger foci observed upon HS (nSBs). Scale bars: 10 μm. The inset shows a magnified view of the corresponding low-magnification image. Representative images from three independent experiments are shown. (B) Left: violin plot of MED26 fluorescence colocalized with Coilin. Right: violin plot of Coilin size (Cajal body size). Vertical line indicates the median. The *p* value was calculated by Welch’s *t* test using R. ∗*p* < 0.05; ∗∗∗∗*p* < 0.0001. Each data point represents an individual cell (NT, *n* = 88 cells; HS, *n* = 93 cells). The experiment was independently repeated three times with similar results. (C) LEC proteins (upper box) and nSB proteins (lower box) found to interact with MED26 under NT and HS conditions in FLAG-MED26 cells, identified by MS. LEC components are listed in ascending order of HS/NT score, indicating weaker interactions with MED26 under HS; nSB components are listed in descending order, indicating increased interactions with MED26 under HS. The IP-MS experiment was independently repeated three times. (D) Volcano plot of SILAC-MS using TurboID-HSF1, showing interactions between HSF1 and multiple Mediator complex subunits enriched under HS vs. NT. Subunits with log2 fold change (log2FC) >1 are highlighted in red. SILAC-MS was performed with three independent biological replicates for each condition (*n* = 3). (E) STRING network analysis of proteins identified by SILAC-MS using TurboID-HSF1 (D). (F) Heatmap of the ChIP signal and gene expression based on our MED1-ChIP-seq, MED26-ChIP-seq, Pol II-ChIP-seq, and RNA-seq data. Genes upregulated upon HS treatment were defined as heat shock response (HSR) genes or putative HSR (Pu-HSR) genes, and 271 genes (padj <0.05, log2FC > 2, baseMean >10) were applied to the analysis. Using publicly available deposited HSF1 ChIP-seq data (GSM4604716 and GSM4604832), genes with an HSF1 peak within 5 kb of their transcription start site (TSS) were classified as Near or HSR genes, whereas genes whose TSSs were located more than 5 kb from the nearest HSF1 peak were classified as Distant or Pu-HSR genes. *n* represents independent biological replicates (ChIP-seq, *n* = 2; RNA-seq, *n* = 3). (G) Left: RNA-FISH images showing colocalization of MED26 (green) with HSF1 (cyan) and HSATIII lncRNA (magenta) under HS. Scale bars: 15 μm. The inset shows an independently acquired representative image. Right: bubble plot showing the fluorescent intensity of HSATIII lncRNA and MED26. (H) RNA-FISH images of HSATIII lncRNA (magenta), MED26 (green), and AFF4 (cyan), showing translocation of MED26 and AFF4 to nSBs under HS. Scale bars: 10 μm. The inset shows an independently acquired representative image. (I and J) IF images of HSF1 (magenta) with Pol II (cyan; I) and ICE1 (green; J). Scale bars: 10 μm. The inset in (I) shows an independently acquired representative image, whereas the inset in (J) shows a magnified view of the corresponding low-magnification image. Line plots of fluorescent intensity are shown to the right of the corresponding images in (H)–(J). The imaging experiments in (G)–(J) were independently repeated three times with similar results, and representative images are shown.

To capture proteins that interact with HSF1 under heat-stress conditions, we first established a cell line using the CRISPR/Cas9 system to knock in HA-tag and FKBP degron to the endogenous HSF1 locus in HCT116 cells to obtain degradation TAG (dTAG)-HSF1 cells (Figure S1B). This design allows the rapid degradation of endogenous HSF1 upon dTAG treatment.^45^ We confirmed that HA-FKBP-HSF1 was degraded upon the addition of dTAG (Figure S1B). We then generated dTAG-HSF1 cells stably expressing TurboID-HSF1 by lentiviral transduction (Figures S1C and S1D). In this system, endogenous HSF1 can be degraded in a dTAG-dependent manner, while TurboID-HSF1 is expressed ectopically, allowing proteins in close proximity to TurboID-HSF1 to be biotinylated upon biotin treatment.^46^ This TurboID-HSF1 cell line was then subjected to stable isotope labeling with amino acids in cell culture (SILAC) to enable relative quantification of proteins proximal to TurboID-HSF1. Upon biotin addition, proteins located within approximately 10 nm of TurboID-HSF1 were biotinylated and subsequently enriched using avidin beads. Mass spectrometric analysis identified multiple Mediator subunits, including MED26 (Figure 1D), thereby supporting the association of HSF1 with the MED26-containing Mediator complex during heat stress. A Search Tool for the Retrieval of Interacting Genes/Proteins (STRING) network analysis using our SILAC-MS data showed that, in addition to RNA Pol II preinitiation complex (including the Mediator complex), proteins proximal to HSF1 upon heat stress were enriched for pathways involved in RNA processing and regulation of TP53 degradation (Figure 1E). In addition, we performed RNA sequencing (RNA-seq), MED26-chromatin immunoprecipitation sequencing (ChIP-seq), MED1-ChIP-seq, and Pol II-ChIP-seq under normal temperatures and heat stress and compared these ChIP-seq peaks with HSF1 ChIP-seq peaks. We found that MED26, MED1, and Pol II signals increased markedly under heat stress at the promoters of genes near HSF1 peaks (Figures 1F and S1E; Table S1). Based on these analyses, we defined genes with increased Mediator and Pol II peaks located near HSF1 peaks, whose transcription start sites (TSSs) were within 5 kb of HSF1 peaks, as HSR genes, and genes with moderately increased Mediator and Pol II peaks located distant from HSF1 peaks, whose TSSs were more than 5 kb away from HSF1 peaks, as putative HSR (Pu-HSR) genes.

During the HSR phase, nSBs composed of HSF1 and HSATIII lncRNAs function as molecular sponges, thereby capturing and protecting mainly spliceosome-associated proteins.^16^ In the subsequent heat stress recovery phase, nSBs are thought to serve as reaction hubs for SRSF phosphorylation, while m^6^A writers (e.g., YTHDC1) are recruited to nSBs to induce m^6^A modification of HSATIII lncRNAs.^16^^,^^18^^,^^20^ To determine whether MED26-localizing foci correspond to nSBs, we performed RNA-fluorescence *in situ* hybridization (FISH) using an HSATIII lncRNA probe. This revealed colocalization of MED26, HSATIII lncRNA, and HSF1 with large foci under heat stress, consistent with MED26 recruitment to nSBs (Figure 1G). Furthermore, while Pol II and the SEC component AFF4 also colocalized with MED26-containing nSBs under heat-stress conditions, the LEC component interactor of LEC ELL subunits (ICE1) did not (Figures 1H–1J). These results suggest that MED26 and SEC are recruited to nSBs to participate in HSATIII lncRNA transcription rather than being passively sequestered. IF using antibodies against additional Mediator subunits showed that, under heat stress, in addition to MED26, MED8, MED12, MED17, and CDK8 are recruited to nSBs (Figures S1F and S1G). MED26 belongs to the middle module of the Mediator complex, whereas MED8 and MED17 are components of the head module, and MED12 and CDK8 belong to the kinase module (Figure S1G). The presence of multiple Mediator modules suggests that MED26 is not recruited to nSBs alone; rather, a MED26-containing Mediator complex as a whole may be recruited to nSBs. These results suggest that MED26-Mediator dissociates from CBs and subsequently colocalizes with HSF1 in nSBs.

### Both the RD and TAD within IDR of HSF1 are necessary and sufficient to recruit the MED26-containing Mediator to artificial HSF1 foci

Next, we investigated the mechanism by which MED26 is recruited to nSBs. Given that HSATIII lncRNAs and HSF1 serve as scaffold factors in nSB assembly,^18^^,^^19^ MED26-Mediator could be recruited to nSBs through direct or indirect interactions with either of these factors. Comprehensive identification of RNA-binding proteins by MS (ChIRP-MS) has previously revealed that the Mediator complex does not copurify with HSATIII,^19^ indicating that MED26 is not recruited by HSATIII lncRNAs. To test this, we performed RNA-FISH after knockdown of HSATIII lncRNA using antisense oligos (ASO) in HeLa cells. Consistent with previous findings,^47^ HSF1 foci were slightly reduced in size by ASO. Similarly, HSATIII lncRNA knockdown had a minimal effect on the colocalization of MED26 with HSF1 foci (Figure S2A), further arguing against HSATIII lncRNA-mediated recruitment of these factors. Additionally, MED1-containing clusters have been shown to co-phase with HSF1 clusters *in vitro*,^10^ supporting the possibility of HSF1-mediated recruitment. To analyze the interaction between HSF1 and MED26, we employed the LacO-LacI system (Figure S2B). Notably, HSATIII lncRNA signals at the LacO-HSF1 foci were barely detectable (Figure S2C), suggesting that factors localized to LacI-HSF1 foci are likely recruited through protein-protein interactions. MED26 was recruited to artificial LacI-HSF1 foci under both normal temperatures and heat-stress conditions (Figure S2D), whereas HSF1 recruitment to LacI-MED26 foci was not detected. In contrast, AFF4, used as a positive control, was efficiently recruited to LacI-MED26 foci (Figure S2E). These results suggest that MED26-Mediator recruitment to nSBs is mediated by HSF1 together with Mediator components other than MED26, rather than by the direct recruitment of HSF1 to MED26. LacI-HSF1 mutants were constructed to explore which of the five domains of human HSF1 mediate its interaction with Mediator complex: DBD, HR-A/B, IDR RD, HR-C, or TAD (Figure 2A).^4^ IF of single-domain deletion mutants using the LacO-LacI system showed minimal colocalization of MED26 and LacI-HSF1 in HSF1 mutants lacking RD or TAD (Figures 2B and 2C and S2F), and deletion of HR-A/B or HR-C had little effect on their colocalization (Figure S2G). In addition, deletion of HSF1 RD or TAD reduced colocalization of the SEC subunit AFF4 (Figure S2G). These results suggest that SEC is recruited to target genes by factors other than MED26. To further evaluate whether the RD and TAD of HSF1 are necessary and sufficient for its interaction with MED26-Mediator, we constructed LacI-HSF1 mutants containing RD, TAD, or RD and TAD (Figure 2A). Indeed, IF analyses of these mutants showed that efficient recruitment of MED26 to the LacO-LacI-HSF1 foci required both RD and TAD (Figures 2D and 2E). Additionally, Mediator subunits MED1, MED12, and MED17 exhibited a similar pattern to that of MED26, indicating that the Mediator complex is recruited to HSF1 (Figure S2H) and that both RD and TAD of HSF1 play a role in this recruitment.

**Figure 2:**
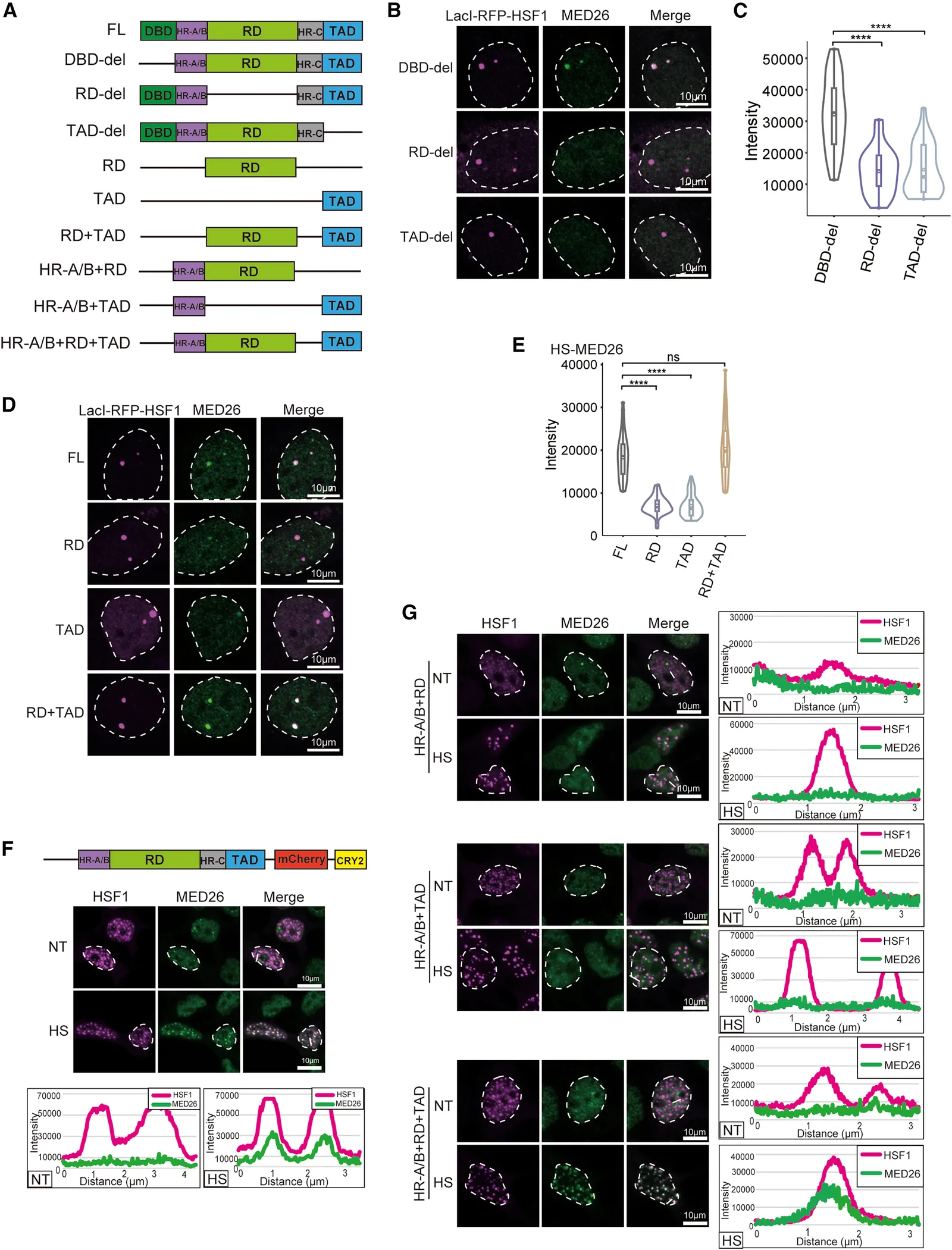
Both RD and TAD (IDR) of HSF1 are required for its interaction with MED26-Mediator (A) Domain structure of full-length (FL) HSF1 and mutants used here. (B) IF images of LacI-RFP-HSF1 mutants (magenta) and MED26 (green), showing minimal colocalization of intrinsic MED26 with HSF1 mutants lacking either RD (RD-del) or TAD (TAD-del) at LacO loci. Scale bars: 10 μm. (C) Violin plot of the fluorescence intensity of MED26 colocalized with LacI-HSF1 mutants in (B). Each data point represents an individual MED26 focus (DBD-del, *n* = 36 foci; RD-del, *n* = 38 foci; TAD-del, *n* = 25 foci). Vertical line indicates the median. *p* values were calculated by Welch’s *t* test using R. ∗∗∗∗*p* < 0.0001. (D) IF images of LacI-RFP-HSF1 (magenta) and MED26 (green) using only RD, TAD, or RD plus TAD HSF1, showing efficient colocalization of MED26 in the presence of both RD and TAD. Scale bars: 10 μm. (E) Violin plot of fluorescent intensity of MED26 colocalized with LacI-HSF1 in (D). Each data point represents an individual MED26 focus (FL, *n* = 64 foci; RD, *n* = 54 foci; TAD, *n* = 49 foci; RD + TAD, *n* = 47 foci). Vertical line indicates the median. *p* values were calculated by Welch’s *t* test using R. ∗∗∗∗*p* < 0.0001; ns, not significant. (F) Upper: schematic of HSF1-DBD-del constructs fused to mCherry-CRY2. Middle: IF images of HSF1-DBD-del (magenta) and MED26 (green) using the optoDroplet system under a NT and HS with light exposure. Scale bars: 10 μm. Lower: line plots of the fluorescence intensity of HSF1 (magenta) and MED26 (green), showing colocalization of MED26 with HSF1 droplets during HS. (G) IF images (left) and line plots of fluorescence intensity (right) in optoDroplet experiments using CRY2-HSF1 mutants (magenta) and MED26 (green) under NT and HS with light exposure, showing colocalization of MED26 with HSF1 foci including both RD and TAD, consistent with results shown in (D). Scale bars: 10 μm. The imaging experiments were independently repeated three times with similar results, and representative images are shown (B–G).

Considering that both the RD and TAD of HSF1 are found within the IDR, essential for foci formation (Figure S3A), we employed the light-inducible optoDroplet system to determine whether MED26 recruitment depends on its ability to form foci. To enable the formation of HSF1 artificial foci upon exposure to light (Figure S3B), we fused HSF1 with mCherry and CRY2 (Figure 2F). Because overexpression of HSF1-CRY2 carrying the HSF1 DBD interfered with artificial foci formation in this system, probably through binding to DNA (Figure S3C), we fused CRY2 with a DBD-deletion (del) mutant of HSF1. After determining that heat stress induced the recruitment of MED26 to artificial HSF1 foci (Figures 2F and S3F), we constructed single-domain deletion mutants of HSF1. This revealed that the trimer formation domain HR-A/B is necessary for artificial foci formation (Figure S3D). Although a previous study has demonstrated that the HR-A/B and RD regions are required for HSF1 condensate formation,^10^ all the HSF1 mutants examined in our optoDroplet system, including the mutant lacking the RD, retained the ability to form artificial foci upon light exposure. Subsequent IF analysis using the optoDroplet system confirmed the importance of RD and TAD in recruiting MED26, showing minimal MED26 colocalization with artificial HSF1 foci containing either domain alone but robust colocalization with HSF1 mutant containing both domains (Figure 2G), consistent with results observed in the LacO-LacI system. Mediator subunit MED1 and SEC subunit AFF4 were also recruited to these artificial HSF1 foci (Figure S3E). These results demonstrate that the two domains (RD and TAD) in the HSF1 IDR are both necessary and sufficient for the recruitment of the MED26-Mediator complex to artificial HSF1 foci.

### A phosphomimetic mutant of the HSF1 RD-TAD region promotes nSB-like foci formation and recruitment of MED26-containing Mediator to these foci

Changes in charge patterning in IDRs, such as phosphorylation, can alter the global conformation of intrinsically disordered proteins, thereby facilitating their selective partitioning into clusters.^47^^,^^48^ Furthermore, many different heat shock-induced post-translational modifications of HSF1 IDRs have been reported, particularly phosphorylation.^10^^,^^11^^,^^12^^,^^49^ We hypothesized that the observed recruitment of MED26-Mediator to HSF1 artificial foci specifically under heat stress in this study depends on phosphorylation of the negatively charged HSF1 IDRs. To test this, we replaced all serine (S) residues in these IDRs (from RD to TAD) with alanine (A) to mimic an unphosphorylated state. Serine-to-aspartate (S > D) and threonine-to-glutamate (T > E) mutations were introduced to mimic phosphorylation within the IDRs (Figure 3A). There was little difference in the intracellular expression levels of the CRY2-mCherry-fused phosphomimetic HSF1 mutants, S > D and T > E, and the non-phosphorylatable HSF1 mutant, S > A (Figure S3F). IF analysis of these mutants in the optoDroplet system revealed decreased colocalization of MED26 with HSF1 S > A mutant foci compared with that of wild-type (WT) HSF1 under heat stress. In contrast, MED26 colocalization was much higher with HSF1 S > D T > E mutant foci than with WT HSF1 under both normal temperatures and heat stress (Figure 3B). RD phosphorylation reportedly promotes LLPS of HSF1 *in vitro*,^10^ and we found that the phosphorylated mimic mutant similarly formed larger artificial foci than those for the WT or S > A mutant using the optoDroplet system (Figure 3C). Furthermore, while the S > A mutant lost its foci-forming ability in the prior study,^10^ our results showed that it retained that ability with CRY2 assistance under heat stress and light exposure, with a lower level of MED26 recruitment to these foci (Figure 3B). Overexpressed proteins may exhibit behaviors that do not reflect their normal biological functions, and this issue is particularly important when overexpressing proteins that contain IDRs or proteins that are incorporated into large complexes, such as the Mediator; however, these results suggest that the recruitment of MED26 depends on HSF1 foci formation and phosphorylation-induced charge alterations in HSF1 IDRs during heat stress.

**Figure 3:**
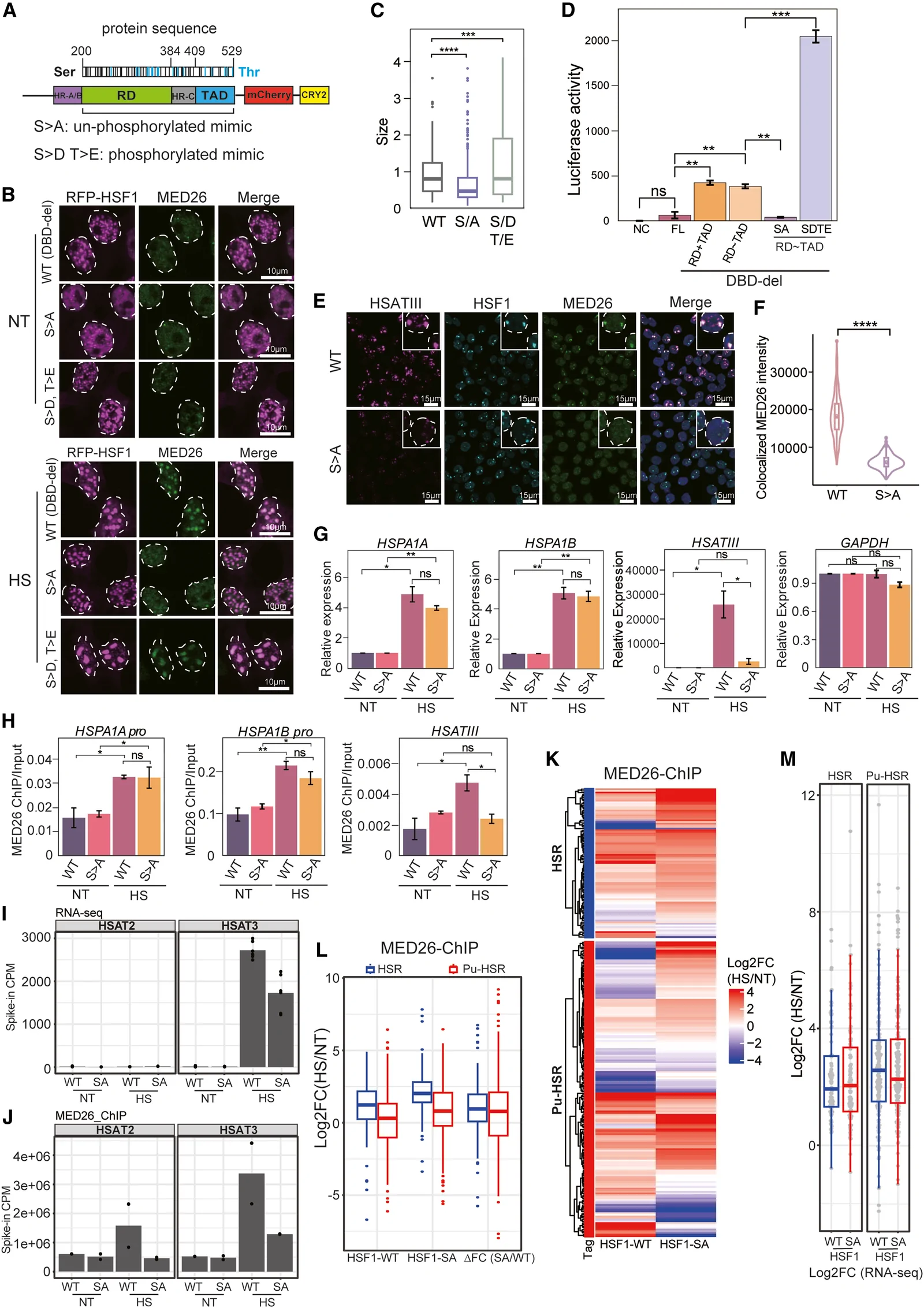
Heat shock-induced hyperphosphorylation of RD-TAD is critical for HSF1 droplet formation and MED26-Mediator recruitment (A) Schematic of HSF1-DBD-del mutants fused to mCherry-CRY2. Serine residues (black) and threonine residues (blue) are shown above the schematic. S > A mutation from RD to TAD mimics unphosphorylated HSF1; S > D and T > E mutations mimic phosphorylated HSF1. (B) IF images of mutant HSF1-CRY2 (magenta) and MED26 (green) in optoDroplet experiments of HEK293T cells with light exposure, showing slight colocalization of MED26 with WT and S > A HSF1 and more prominent colocalization with S > D T > E HSF1 under a normal temperature (NT; upper), in contrast to barely detectable MED26 colocalization with S > A HSF1 and strong colocalization with WT and S > D T > E HSF1 under heat shock (HS; lower). Scale bars: 10 μm. The imaging experiments were independently repeated three times with similar results, and representative images are shown. (C) Boxplot of CRY2-HSF1 focus size during HS with light exposure. Each data point represents an individual HSF1 focus (WT, *n* = 197 foci; S > A, *n* = 365 foci; S > D T > E, *n* = 212 foci). *p* values were calculated by Welch’s *t* tests using R. ∗∗∗*p* < 0.001; ∗∗∗∗*p* < 0.0001. (D) Fluorescence intensity in luciferase reporter assays of HSF1 mutants under NT conditions. Data represent three independent experiments (*n* = 3), each performed in triplicate. *p* values were calculated by pairwise two-tailed Student’s *t* tests using R. ∗∗*p* < 0.01; ∗∗∗*p* < 0.001; ns, not significant. (E) RNA-FISH images of HSATIII (magenta), HSF1 (cyan), and MED26 (green) in cells stably expressing HSF1-WT and S > A mutant HSF1, showing MED26 accumulation at nSBs and decreased expression of HSATIII in S > A-expressing cells. Nuclear DNA was stained with DAPI (blue). Scale bars: 15 μm. The inset shows an independently acquired representative image. The imaging experiments were independently repeated three times with similar results, and representative images are shown. (F) Violin plot of MED26 fluorescence intensity colocalized with HSF1 in cells stably expressing HSF1-WT and S > A mutant shown in (E). Each data point represents an individual MED26 focus (WT, *n* = 215 foci; S > A, *n* = 318 foci). The experiment was independently repeated three times with similar results. *p* values were calculated by Welch’s *t* tests using R. The experiments in (E) and (F) were performed under HS conditions. ∗∗∗∗*p* < 0.0001. (G) RT-qPCR analysis of *HSPA1A*, *HSPA1B*, *HSATIII*, and *GAPDH* expressions in dTAG-HSF1 cells rescued with HSF1-WT and HSF1 S > A under NT and HS conditions. Calculated by unpaired two-tailed Student’s *t* test (*n* = 3 independent biological replicates): ns, not significant; ∗*p* < 0.05; ∗∗*p* < 0.01; ∗∗∗*p* < 0.001; ∗∗∗∗*p* < 0.0001. (H) MED26-ChIP-qPCR analysis of the HSPA1A and HSPA1B promoters and HSATIII using HSF1-WT and HSF1-S > A rescued cells under NT and HS conditions. As calculated by unpaired two-tailed Student’s *t* test (*n* = 4 independent biological replicates): ns, not significant; ∗*p* < 0.05; ∗∗*p* < 0.01; ∗∗∗*p* < 0.001; ∗∗∗∗*p* < 0.0001. (I) Bar plot showing HSAT2 and HSAT3 read counts from our RNA-seq data for rescued cells stably expressing HSF1-WT and HSF1-S > A under NT and HS conditions. The results also show high HSAT3 detection sensitivity. CPM values were normalized using *Drosophila* spike-in reads (*n* = 3 independent biological replicates). (J) Bar plot showing the HSAT2 and HSAT3 read counts from our MED26-ChIP-seq data using HSF1-WT and HSF1-S > A cells under NT and HS conditions. CPM values were normalized using *Drosophila* spike-in reads (*n* = 2 independent biological replicates). (K) Heatmap of the ChIP signal in HSR genes and Pu-HSR genes based on our MED26-ChIP-seq data using HSF1-WT and HSF1-S > A stably expressing cell lines (*n* = 2 independent biological replicates). (L) Boxplot of the change in MED26 ChIP signal (log2 fold change [HS/NT] values) from MED26-ChIP-seq data using HSF1-WT and HSF1-S > A cells (*n* = 2 independent biological replicates). (M) Boxplot showing the expression change (log2 fold change [HS/NT]) based on our RNA-seq analyses of HSF1-WT and HSF1-S > A cells (*n* = 3 independent biological replicates).

Next, we performed luciferase assays to evaluate the transcriptional activity of HSF1 mutants (Figure S3G). In particular, we fused the GAL4 DBD, which binds to the Upstream Activating Sequence (UAS) of the luciferase reporter gene, to each HSF1 construct. Constructs retaining transactivation activity, such as full-length HSF1, are expected to recruit transcriptional coactivators, including the Mediator complex, and activate luciferase transcription. Thus, luciferase activity serves as a readout of HSF1-dependent transcriptional activity. Since luciferase enzymatic activity can be affected by heat shock, this assay was performed under non-HS conditions. Consistently, the S > D T > E mutant showed greater transcriptional activity than that of WT HSF1, and the unphosphorylated mimic S > A mutant exhibited almost no transcriptional activity (Figure 3D). Furthermore, neither of the HSF1 RD and TAD deletion mutants showed any transactivation activity (Figure S3H).

Given the artificial nature of the LacI-LacO and optoDroplet systems and the caveats that overexpression may lead to non-physiological behavior, we examined the effect of HSF1 mutants on the HSR under physiological conditions. Endogenous HSF1 was depleted using the dTAG system, and WT HSF1 or the SA mutant was re-expressed in a doxycycline (Dox)-inducible manner in HCT116 cells using a lentiviral expression system. In the presence of dTAG, endogenous HSF1 was efficiently degraded, and Dox treatment induced robust expression of WT HSF1. In contrast, in cells expressing the HSF1 SA mutant, leaky expression was detectable even in the absence of Dox; however, Dox treatment strongly induced expression of the HSF1 SA mutant. In addition, a marked upward mobility shift of WT HSF1 was observed specifically under heat-stress conditions. Because this shift was not observed in the S > A mutant, it is likely attributable to heat stress-induced phosphorylation of HSF1 (Figure S3I and S3J). Using these cell lines, we performed RNA-FISH under heat stress. Using a probe targeting HSATIII lncRNA, HSATIII lncRNA expression was substantially lower in S > A-expressing cells than in WT cells (Figures 3E and 3F). Colocalization of MED26 with nSBs was also markedly decreased in S > A cells, indicating that RD-TAD phosphorylation is required for the efficient recruitment of MED26-Mediator to activate transcription of HSATIII lncRNA (Figure 3E).

We performed quantitative reverse-transcription PCR (RT-qPCR) and RNA-seq analyses using these dTAG and Dox-inducible rescue cell lines to evaluate global changes in gene expression. While the expression levels of HSP genes and *GAPDH* were comparable between the HSF1 S > A mutant-expressing and WT cells (Figures 3G and 3M), *HSATIII* expression was greatly decreased in S > A mutant cells (Figure 3G). For the RNA-seq analysis of HSATIII RNAs, we quantified reads containing HSATIII repeat motifs (ATTCC, TTCCA, TCCAA, CCAAT, or CAATT) from the sequenced reads, providing a basis for distinguishing HSATIII from HSATII (Figures S3K and S3L). Consistent with RT-qPCR results, *HSATIII* expression was decreased in S > A mutant cells (Figure 3I). Next, we performed ChIP-seq to examine MED26 occupancy at those genes. In HSF1 S > A mutant-expressing cells, MED26 occupancy at HSP genes did not differ from that in WT cells, whereas occupancy at *HSATIII* genes was markedly reduced (Figure 3H). MED26 ChIP-seq signals showed little difference between cells expressing WT HSF1 and those expressing the S > A mutant at both HSR and Pu-HSR gene groups (Figures 3K and 3L). Similarly, heat shock-induced changes in HSR and Pu-HSR gene groups were largely comparable between WT HSF1- and S > A mutant-expressing cells (Figure 3M). These findings indicate that phosphorylation of the RD and TAD of HSF1 is necessary for efficient recruitment of MED26 to *HSATIII* genes, with a greater effect on the expression of *HSATIII* lncRNA than on the transcription of HSR genes.

### MED15 is responsible for MED26-Mediator colocalization with nSBs

Based on the observed role of HSF1 RD-TAD phosphorylation, we further investigated the interaction between HSF1 and MED26-Mediator. MED26 contains an NTD that interacts with the transcription factor IID complex, SEC, or LEC; a C-terminal domain (CTD) that interacts with the Mediator complex and Pol II; and a large IDR between the NTD and CTD.^27^^,^^35^^,^^36^^,^^37^ To determine the MED26 domain required for the interaction with HSF1, we transiently coexpressed LacI-red fluorescent protein (RFP)-HSF1 with enhanced green fluorescent protein (GFP)-tagged MED26 mutants in U2OS cells. Five GFP-tagged MED26 variants were generated: NTD-del, IDR-del, IDR alone, CTD-del, and Mediator docking site (10 amino acids of CTD)-del (Figures 4A and S4A). MED26 mutants lacking the CTD were not recruited to LacI-LacO-HSF1 foci, indicating that the CTD is required for MED26 recruitment to HSF1 in this system. Notably, deletion of the 10 amino acid Mediator-interaction region within the CTD was sufficient to abolish recruitment (Figure 4B). This result raised the possibility that HSF1 recruits MED26 through interactions with other components of the Mediator complex.

**Figure 4:**
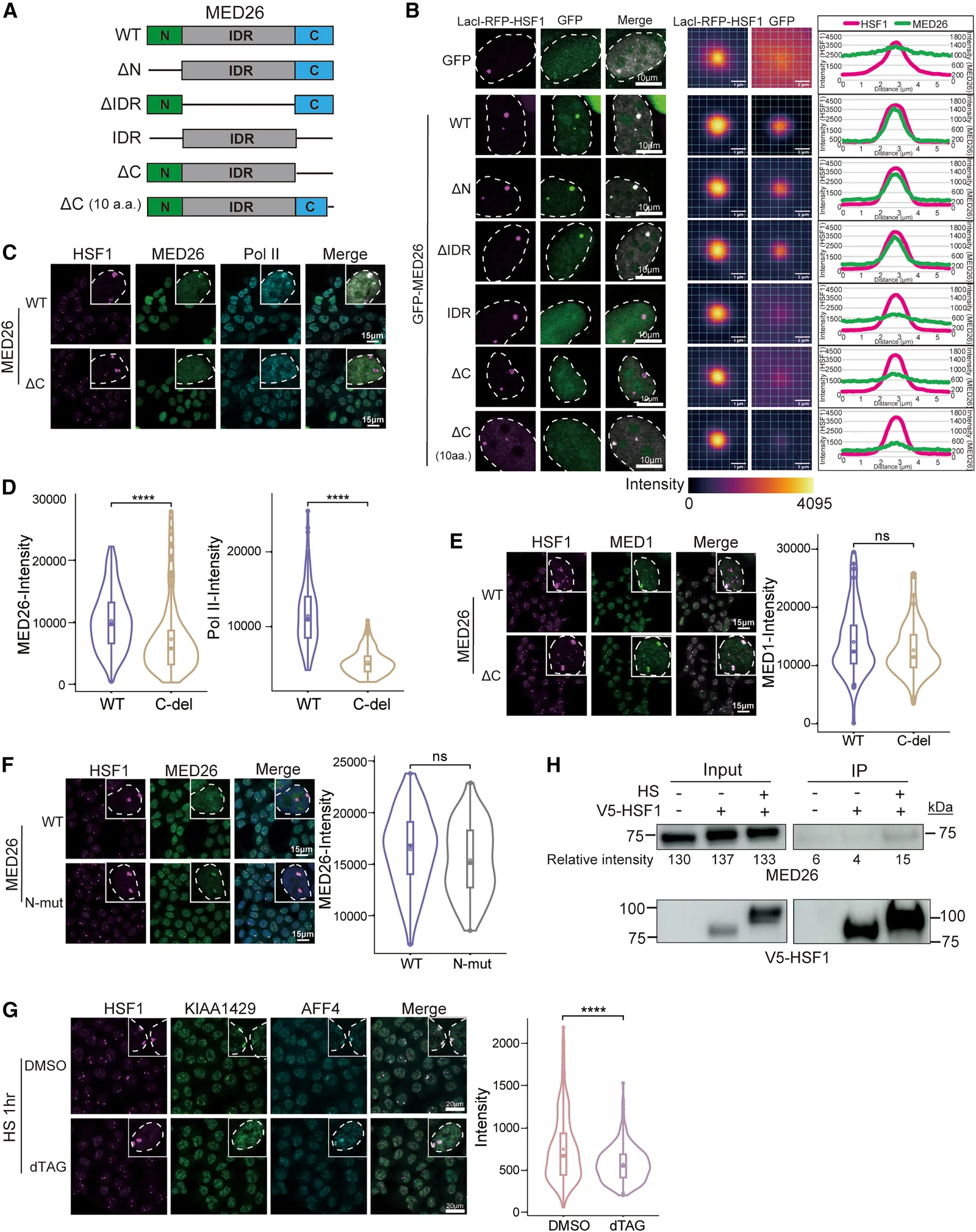
MED15 is necessary for MED26-Mediator colocalization with nSBs (A) Domain structure of MED26 and variants. (B) Left: IF images of LacI-RFP-HSF1 (magenta) and GFP-MED26 mutants (green). GFP without MED26 was used as a negative control. MED26 mutants: ΔN, N-terminal deletion; ΔIDR, IDR deletion; IDR, contains only the IDR; ΔC, C-terminal deletion; ΔC (10 aa), deletion of the 10 amino acids of the CTD that interact with the Mediator complex. Scale bars: 10 μm. Middle: merged images of the corresponding IF staining images, showing differences in GFP fluorescence intensity colocalized with LacI-HSF1 foci. Scale bars: 1 μm. Right: line plots of the merged images. (C) IF images of HSF1 (magenta), MED26 (green), and Pol II (cyan) in the MED26-wild-type (WT) and MED26-ΔC-rescued cells. Scale bars: 15 μm. The inset shows an independently acquired representative image. (D) Violin plot of MED26 and Pol II fluorescent intensity (C) colocalized with HSF1. Each data point represents an individual MED26 and Pol II focus (WT, *n* = 112 foci; ΔC, *n* = 124 foci). *p* values were calculated by Welch’s *t* tests using R. ∗∗∗∗*p* < 0.0001. (E) Left: IF images of HSF1 (magenta) and MED1 (green) in MED26-WT and MED26-ΔC-rescued cells. Scale bars: 15 μm. The inset shows an independently acquired representative image. Right: violin plot of MED1 fluorescent intensity colocalized with HSF1. Each data point represents an individual MED1 focus (WT, *n* = 67 foci; ΔC, *n* = 89 foci). The *p* value was calculated by Welch’s *t* test using R. ns, not significant (*p* = 0.093). (F) Left: IF images of HSF1 (magenta) and MED26 (green) in MED26-WT and MED26 NTD mutant (N-mut)-rescued cells. Scale bars: 15 μm. The inset shows an independently acquired representative image. Right: violin plot of MED26 fluorescent intensity colocalized with HSF1. Each data point represents an individual MED26 focus (WT, *n* = 79 foci; N-mut, *n* = 80 foci). The *p* value was calculated by Welch’s *t* test using R. ns, not significant (*p* = 0.064). (G) Left: IF images of HSF1 (magenta), KIAA1429 (green), and AFF4 (cyan) in dTAG-MED26 cells during a 1-h recovery at 37°C following a 1-h heat shock (HS). Scale bars: 20 μm. The inset shows an independently acquired representative image. Right: violin plot of KIAA1429 fluorescent intensity colocalized with HSF1. Each data point represents an individual KIAA1429 focus (DMSO, *n* = 268 foci; dTAG, *n* = 276 foci). The *p* value was calculated by Welch’s *t* test using R. ∗∗∗∗*p* < 0.0001. The imaging experiments in (B)–(G) were independently repeated three times with similar results, and representative images are shown. (H) Western blot analysis of the co-precipitation of MED26 with HSF1 in HSF1-WT stably expressing cell extracts. The experiment was independently repeated three times with similar results, and a representative blot is shown.

As previously reported, the NTD mutant abolishes the MED26 interaction with the SEC, whereas deletion of 10 amino acids in the CTD abolishes the interaction with the Mediator complex.^27^^,^^35^ To further dissect the domains of MED26 required for recruitment to nSBs, we generated mutant cell lines expressing HA-tagged MED26 NTD mutants (R61A, K62A) using the CRISPR/Cas9 system (Figure S4B). We also generated CTD mutant cell lines while depleting endogenous MED26 using the dTAG system. As shown in Figure S4C, dTAG-MED26 was degraded by adding dTAG. Using the dTAG-MED26 cell line, the MED26-WT or MED26-CTD mutant was exogenously expressed (Figure S4D). IF analysis showed that the MED26 CTD deletion abolished the colocalization of Mediator components MED26, MED1, and Pol II at nSBs (Figures 4C–4E). Consistent with these findings, the CTD deletion mutant significantly reduced the expression of HSATIII lncRNA and *HSP70* (Figure S4E). These results indicated that MED26 binding to the Mediator is critical for the recruitment of Pol II to *HSATIII* and HSP genes and for their transcription upon heat stress. In contrast, the MED26 NTD mutation did not affect MED26 colocalization with HSF1 at nSBs (Figure 4F). Consistent with these results, transcription of HSATIII lncRNA and *HSP70* under heat stress was minimally affected by MED26 NTD-mut-expressing cell lines (Figure S4E). In addition, colocalization of AFF4, a component of SEC at nSBs, was unaffected by MED26 knockdown; however, colocalization of KIAA1429, a representative marker of nSBs, was abolished (Figure 4G). In addition, co-immunoprecipitation followed by western blotting showed that endogenous MED26 co-precipitates with V5-tagged HSF1, which further confirmed that the Mediator complex containing MED26 interacts with HSF1 (Figure 4H).

We tested which Mediator components directly interact with HSF1. We initially focused on MED15 and MED25 based on previous work demonstrating Hsf1 recruitment of MED15 in yeast^38^ and the potential role of MED25 in the *Arabidopsis thaliana* stress response.^50^ Having found that MED15 and MED25 colocalized with nSBs upon heat stress (Figure S4F), we established stable cell lines with knockdown of endogenous MED15 and MED25 using the dTAG system (Figures S4G and S4H). RNA-FISH was then used to investigate the effects of MED15, MED25, and MED26 knockdown on *HSATIII* gene expression and colocalization of HSF1 and MED26 at nSBs (Figure 5A). HSATIII lncRNA expression decreased substantially under MED15 or MED26 knockdown but not under MED25 knockdown (Figure 5A). MED26 enrichment at nSBs was lost under MED15 knockdown (Figures 5A and 5B). To validate the effects of MED15 knockdown on other Mediator subunits, we performed IF using anti-MED1 and anti-MED12 antibodies in cells with MED15 knockdown, thereby revealing disruptions in their recruitment to nSBs (Figure S4I).

**Figure 5:**
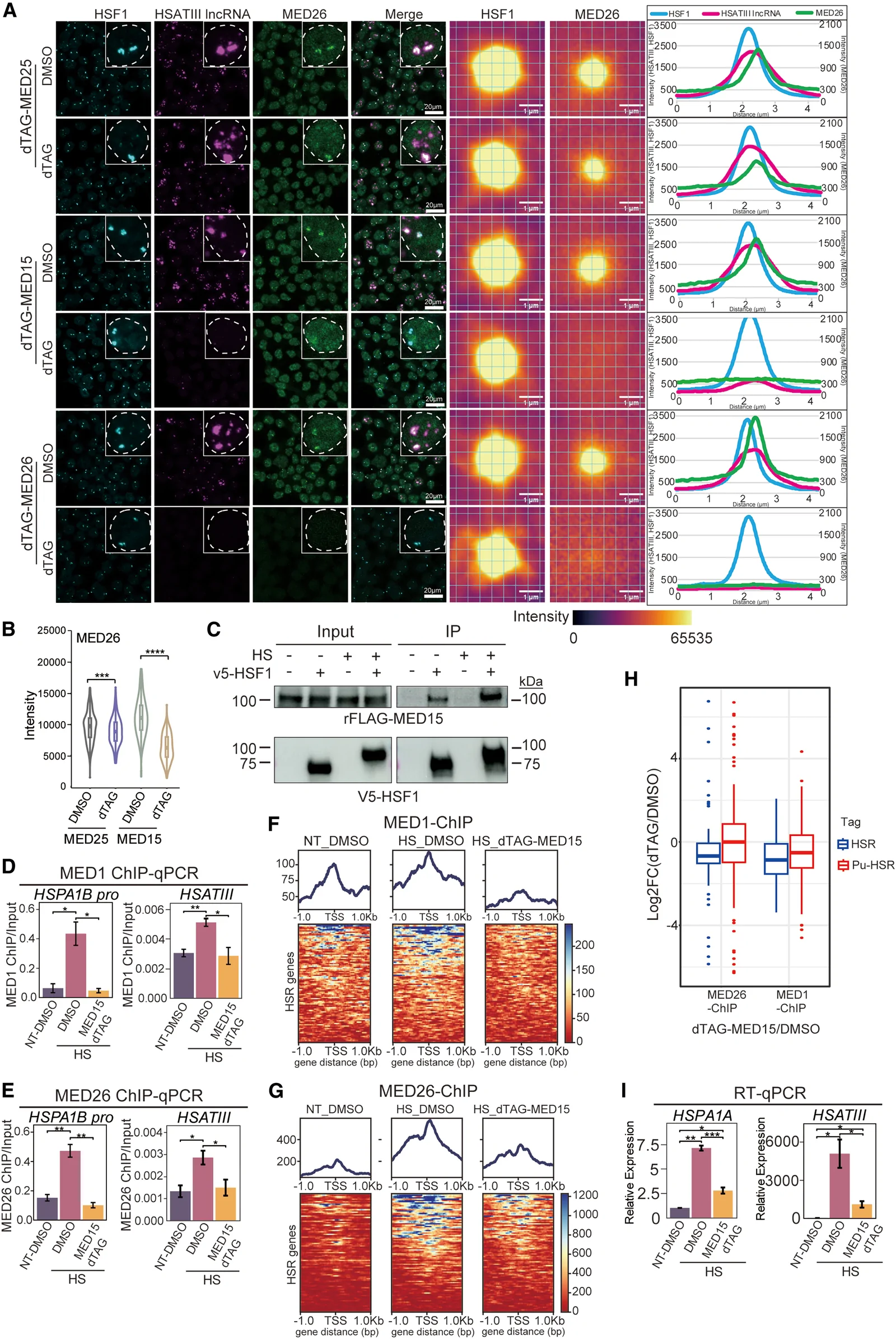
MED15 is required for MED26-Mediator recruitment to HSPs and *HSATIII* genes (A) Left: RNA-FISH images of HSATIII lncRNA (magenta), HSF1 (cyan), and MED26 (green) using MED15, MED25, and MED26 dTAG cell lines. Scale bars: 20 μm. Middle: the averaged signals obtained by integrating HSF1 and MED26 signals that colocalized with HSATIII. The inset shows a magnified view of the corresponding low-magnification image. Right: line plots of HSATIII lncRNA, MED26, and HSF1 fluorescence intensity. The imaging experiments were independently repeated three times with similar results, and representative images are shown. (B) Violin plot of MED26 fluorescence intensity colocalized with HSF1 using dTAG-MED15 and dTAG-MED25 cells, showing decreased colocalization under MED15 knockdown. Each data point represents an individual MED26 focus (DMSO-MED25, *n* = 313 foci; dTAG-MED25, *n* = 292 foci; DMSO-MED15, *n* = 275 foci; dTAG-MED15, *n* = 238 foci). *p* values were calculated by Welch’s *t* test using R. ns, not significant. ∗∗∗*p* < 0.001; ∗∗∗∗*p* < 0.0001. (C) Western blotting to evaluate *in vitro* binding using purified recombinant FLAG-MED15 (rFLAG-MED15) incubated with bead-bound V5-HSF1 prepared from HCT116 cells subjected to normal temperature (NT) or heat-shock (HS) conditions. The binding of FLAG-MED15 to HSF1 was markedly enhanced under HS. The experiment was independently repeated three times with similar results, and a representative blot is shown. (D) Bar plots showing MED1-ChIP-qPCR results for the *HSPA1B* promoter and HSATIII lncRNA in dTAG-MED15 cells under NT and HS conditions. *p* values were calculated by unpaired two-tailed Student’s *t* tests (*n* = 3 independent biological replicates). ns, not significant. ∗*p* < 0.05; ∗∗*p* < 0.01. (E) Bar plots showing MED26-ChIP-qPCR results for the *HSPA1B* promoter and HSATIII lncRNA (left) in dTAG-MED15 cells under NT and HS conditions. *p* values were calculated by unpaired two-tailed Student’s *t* tests (*n* = 4 independent biological replicates). ns, not significant. ∗*p* < 0.05; ∗∗*p* < 0.01. (F) Upper: metagene plot of our MED1-ChIP-seq in dTAG-MED15 cells. Lower: heatmap showing the MED1-ChIP signal in dTAG-MED15 cells; colors indicate signal intensity. Transcription start sites (TSSs) ± 1 kb are shown in plots (*n* = 2 independent biological replicates). (G) Upper: metagene plot of our MED26-ChIP-seq in dTAG-MED15 cells. Lower: heatmap showing the MED26-ChIP signal in dTAG-MED15 cells; colors indicate signal intensity. TSS ±1 kb are shown in plots (*n* = 2 independent biological replicates). (H) Boxplot of the change in MED26-ChIP signal and MED1-ChIP signal (log2 fold change [dTAG/DMSO] values) from MED26-ChIP-seq data and MED1-ChIP-seq data using dTAG-MED15 cells. HSR and Pu-HSR genes were defined in (F) (*n* = 2 independent biological replicates). (I) RT-qPCR analysis of *HSP70* and HSATIII lncRNA expression in dTAG-MED15 cells. *p* values were calculated by unpaired two-tailed Student’s *t* tests (*n* = 4 independent biological replicates). ns, not significant. ∗*p* < 0.05; ∗∗*p* < 0.01; ∗∗∗*p* < 0.001.

To test whether MED15 interacts with HSF1 *in vitro*, FLAG-tagged MED15 purified from Sf9 cells was incubated with V5-tagged HSF1, which were immunoprecipitated from HCT116 cells cultivated under normal temperature or heat-shock conditions. Following extensive washing, bound proteins were analyzed by western blotting. Recombinant FLAG-MED15 interacted with HSF1, and this interaction was increased upon heat shock (Figure 5C). In addition, upon HS, HSF1 exhibited a mobility shift, indicating that HSF1 is highly phosphorylated in response to heat shock (Figure 5C). These results suggest that MED15 directly interacts with HSF1 and that HSF1 phosphorylation promotes this interaction.

Human MED12 reportedly interacts with HSF1 and promotes HSF1 condensate formation at HSP gene promoters.^42^^,^^43^ To confirm whether MED12 contributes to Mediator recruitment to nSBs, we performed IF using MED12-knockdown cells. Acute depletion of MED12 using the dTAG system did not affect the colocalization of MED26 with HSF1 at nSBs (Figures S4J and S4K). Collectively, our results support the conclusion that a direct interaction between MED15 and HSF1 is critical for Mediator complex recruitment to nSBs, despite the lack of amino acid sequence conservation in these factors between humans and yeast^51^ (Figure S5A).

Given that MED15 is necessary for MED26-Mediator recruitment to nSBs, we used ChIP-qPCR to investigate Mediator binding to MED1 and MED26 under MED15 or MED26 knockdown. MED15 knockdown decreased the occupancy of MED1 and MED26 across the *HSPA1B* and *HSATIII* gene regions (Figures 5D and 5E). We further evaluated how acute dTAG-mediated degradation of MED15 affects MED1 and MED26 occupancy at HSR gene regions through a ChIP-seq analysis. MED15 knockdown decreased the occupancy of MED1 and MED26 at HSR genes, particularly at the HSR genes (Figures 5F–5H). Consistent with the results, MED15 knockdown significantly reduced the transcript levels of HSR genes and HSATIII lncRNA (Figure 5I). These findings demonstrate that MED26-Mediator is recruited to HSP and *HSATIII* genes through MED15.

### MED26-dependent assembly of a putative Mediator-Pol II complex at nSBs

To verify the function of MED26 during the HSR, we performed ChIP-seq analyses of MED1 and Pol II using MED26 knockdown cells. MED26 knockdown by dTAG decreased MED1 and Pol II occupancy at HSR genes (Figures 6A, 6D, 6E, S5E, and S5F). The reduction in ChIP signals upon MED26 knockdown was particularly pronounced at HSR gene regions (Figure 6B). Consistently, RNA-seq analysis of dTAG-mediated MED26 knockdown cells showed that HSR gene expression decreased markedly upon dTAG treatment, with a preferential reduction at HSR genes more than Pu-HSR genes (Figures 6C, S5B, and S5C). Next, we examined the effect of MED26 knockdown on HSATIII lncRNA at nSBs. RNA-FISH analysis revealed that HSATIII signals at nSBs were completely abolished by MED26 knockdown, whereas HSF1 still formed nSBs (Figure 6F). Next, we tested whether MED26 knockdown would impact Mediator and Pol II colocalization with HSF1 at nSBs. IF analysis revealed that MED26 knockdown reduced Pol II colocalization with HSF1 but did not affect the colocalization of HSF1 with other Mediator subunits, including MED1 and MED12 (Figures 6G, 6H, and S5D). Consistent with these findings, ChIP-qPCR analysis demonstrated that MED26 knockdown also decreased the occupancy of Pol II, but not MED1, at *HSATIII* genes (Figures 6I and 6J). Quantification of ChIP-seq reads containing HSATIII repeat motifs showed that MED26 knockdown decreased the occupancy of Pol II, but not MED1, at *HSATIII* genes (Figures 6K and 6L). These results align with those of previous studies showing that Mediator complexes containing MED26 have the ability to bind with Pol II.^27^ Upon depletion of MED26, Pol II occupancy was markedly reduced at HSATIII loci and HSP genes, resulting in diminished transcription of both (Figures 6M and 6N).

**Figure 6:**
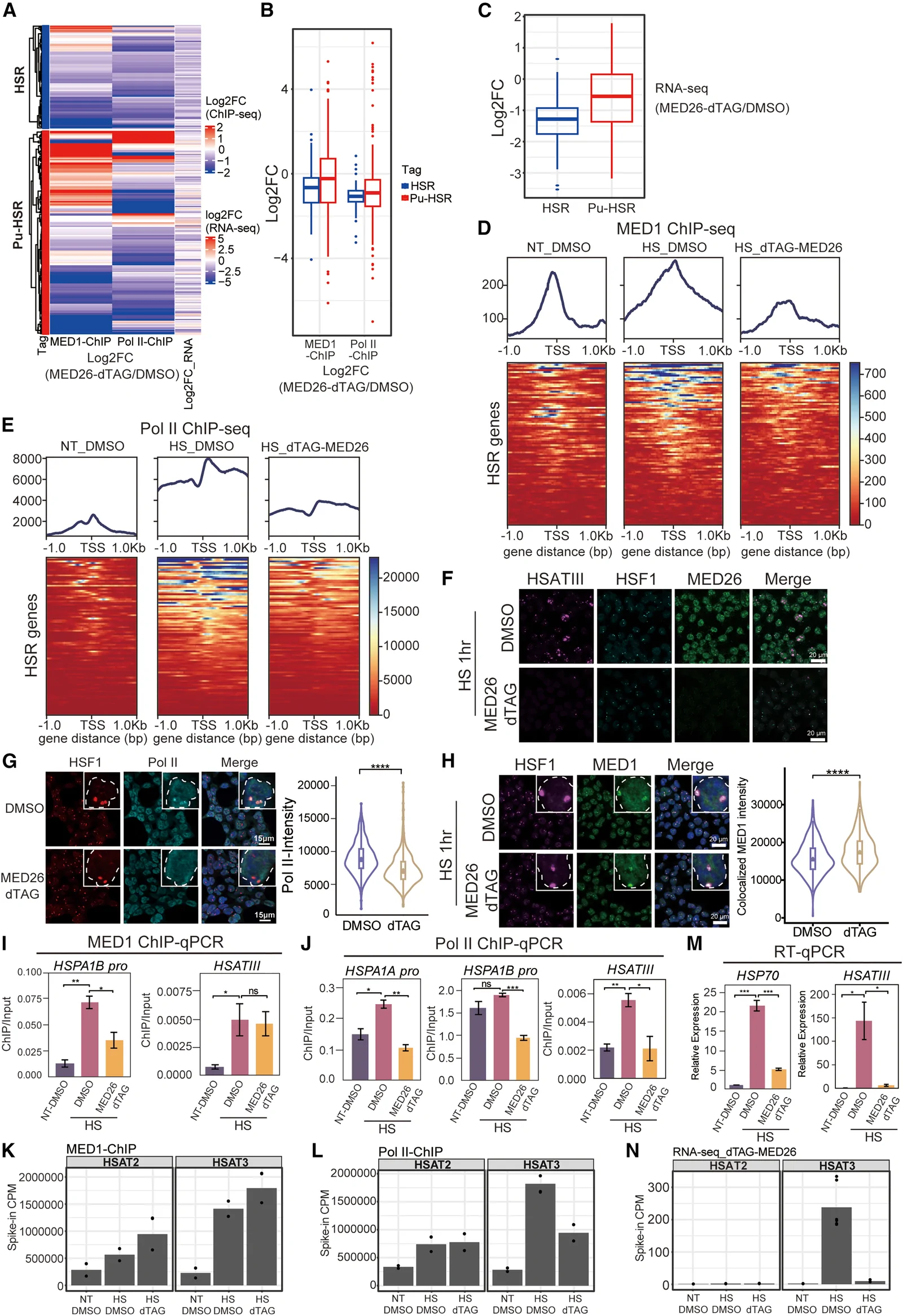
MED26 is required for Pol II recruitment to HSP genes and *HSATIII* genes at nSBs during heat stress (A) Heatmap of the ChIP signal in HSR and Pu-HSR genes based on our MED1-ChIP-seq, Pol II-ChIP-seq, and RNA-seq data using dTAG-MED26 cells (ChIP-seq, *n* = 2 independent biological replicates; RNA-seq, *n* = 3 independent biological replicates). (B) Boxplot of the change in ChIP signal (log2 fold change [MED26-dTAG/DMSO] values) from MED1-ChIP-seq and Pol II-ChIP-seq data using dTAG-MED26 cells (*n* = 2 independent biological replicates). (C) Boxplot showing the expression change (log2 fold change [MED26-dTAG/DMSO]) based on our RNA-seq using dTAG-MED26 cells (*n* = 3 independent biological replicates). (D) Upper: metagene plot of our MED1-ChIP-seq in dTAG-MED26 cells. Lower: heatmap showing the MED1-ChIP signal in dTAG-MED26 cells; colors indicate signal intensity. Transcription start sites (TSSs) ± 1 kb are shown in plots (*n* = 2 independent biological replicates). (E) Upper: metagene plot of our Pol II-ChIP-seq in dTAG-MED26 cells. Lower: heatmap showing the Pol II-ChIP signal in dTAG-MED26 cells. TSS ±1 kb are shown in plots (*n* = 2 independent biological replicates). (F) FISH images of HSATIII lncRNA (magenta), HSF1 (cyan), and MED26 (green) in dTAG-MED26 cells exposed to HS, showing significantly decreased HSATIII lncRNA levels under MED26 degradation. Scale bars: 20 μm. The experiments were independently repeated three times with similar results, and representative images are shown. (G) Left: IF images of HSF1 (red) and Pol II (cyan) in cells under MED26 knockdown and HS, showing decreased colocalization of Pol II with HSF1 in nSBs. Scale bars: 15 μm. The inset shows a magnified view of the corresponding low-magnification image. Right: violin plot of Pol II fluorescent intensity colocalized with HSF1 upon HS. Each data point represents an individual Pol II focus (DMSO, *n* = 461 foci; dTAG, *n* = 449 foci). ∗∗∗∗*p* < 0.0001. The imaging experiments were independently repeated three times with similar results, and representative images are shown. (H) Left: IF images of HSF1 (magenta) and MED1 (green) in cells under MED26 knockdown and HS. Scale bars: 20 μm. The inset shows a magnified view of the corresponding low-magnification image. Right: violin plot of MED1 fluorescent intensity colocalized with HSF1 upon HS. Each data point represents an individual MED1 focus (DMSO, *n* = 304 foci; dTAG, *n* = 229 foci). ∗∗∗∗*p* < 0.0001. The imaging experiments were independently repeated three times with similar results, and representative images are shown. (I) Bar plots showing MED1-ChIP-qPCR results for the *HSPA1B* promoter and HSATIII lncRNA. ns, not significant. ∗*p* < 0.05; ∗∗*p* < 0.01, calculated by unpaired two-tailed Student’s *t* tests (*n* = 5 independent biological replicates). (J) Bar plots showing Pol II-ChIP-qPCR results for the HSPA1A and HSPA1B promoters and HSATIII lncRNA. ns, not significant. ∗*p* < 0.05; ∗∗*p* < 0.01; ∗∗∗*p* < 0.001, calculated by unpaired two-tailed Student’s *t* tests (*n* = 4 independent biological replicates). (K) Bar plot showing the HSAT2 and HSAT3 read counts from our MED1-ChIP-seq data using dTAG-MED26 cells (*n* = 2 independent biological replicates). (L) Bar plot showing the HSAT2 and HSAT3 read counts from our Pol II-ChIP-seq data using dTAG-MED26 cells (*n* = 2 independent biological replicates). (M) Bar plots showing RT-qPCR results for HSP70 and HSATIII lncRNA expression. ns, not significant. ∗*p* < 0.05; ∗∗*p* < 0.01; ∗∗∗*p* < 0.001, calculated by unpaired two-tailed Student’s *t* tests (*n* = 4 independent biological replicates). (N) RNA-seq read counts of HSAT2 and HSAT3 in dTAG-MED26 cells under NT and HS conditions (*n* = 3 independent biological replicates). CPM values (K, L, and N) were normalized using *Drosophila* spike-in reads.

In summary, under heat-stress conditions, Mediator complexes containing MED26 are recruited to both HSR gene loci and HSATIII lncRNA-associated nSBs through direct interactions with HSF1 and MED15. Importantly, phosphorylation of HSF1, particularly within its IDR, preferentially enhances the recruitment of the Mediator to nSBs rather than to HSR gene loci. Acute depletion of MED26 results in the loss of both the Mediator and Pol II from HSR gene promoters, whereas the Mediator at HSATIII loci is largely retained despite Pol II dissociation. Further analyses are necessary to clarify this issue, as we cannot exclude the possibility that the observed difference is due to distinct induction kinetics of HSATIII and HSR gene transcription. In addition, ChIP-based measurements of occupancy may differ among Mediator subunits due to differences in antibody performance or epitope accessibility. Collectively, these findings support a model in which MED26 acts as a ligand-like molecular switch during heat stress, stabilizing the Mediator-Pol II complex assembly specifically at nSBs to facilitate nSB formation (Figure 7A).

**Figure 7:**
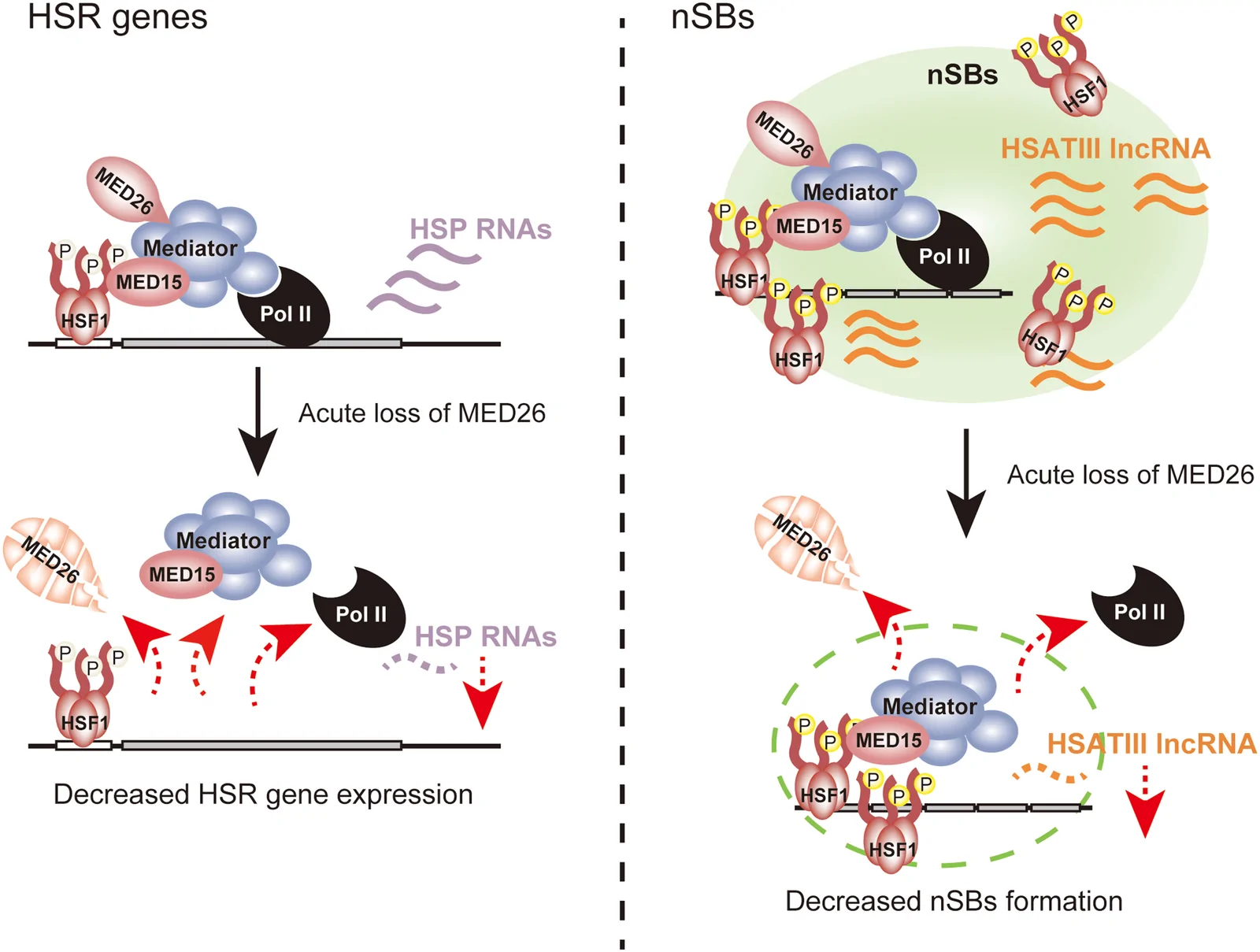
Model of MED26 acting as a molecular switch for Mediator-Pol II assembly at nSBs during heat stress Schematic model for recruitment of MED26-Mediator to both HSR genes and HSATIII gene-associated nSBs via interactions with HSF1 and MED15. Phosphorylation of HSF1, particularly within its IDR, preferentially promotes Mediator accumulation at nSBs rather than at HSR gene loci. Loss of MED26 displaces the Mediator and Pol II from HSR genes but leaves the Mediator at HSATIII genes in nSBs, indicating that MED26 functions as a molecular switch, thereby enabling Mediator binding with Pol II and leading to nSB formation through HSAT III lncRNA transcription.

## Discussion

In this study, we demonstrate that the Mediator complex is recruited to heat stress-responsive loci through the RD-TAD region of HSF1 via MED15 and that MED26 is indispensable for the efficient recruitment of Pol II during the early phase of the HSR in human cell lines. Previous studies have largely focused on the recovery phase, highlighting the sequestration and protection of splicing factors within nSBs and their subsequent roles in post-stress gene regulation,^18^^,^^19^^,^^20^^,^^52^ and the transcriptional regulatory mechanisms operating at the onset of heat stress have remained poorly understood. Our findings address this gap and provide direct evidence that HSF1 engages the Mediator complex to initiate transcriptional responses immediately following heat shock, with MED26 playing a central role in Pol II recruitment.

Our results support a model in which transcriptional regulation during the early HSR is executed through two mechanistically distinct modes: regulation at conventional genomic loci, exemplified by HSR genes, and regulation within nSBs formed at HSATIII loci (Figure 7). Upon heat shock, extensive post-translational modifications, particularly phosphorylation, activate HSF1 and promote its binding to HSEs.^4^^,^^10^^,^^11^^,^^49^ Activated HSF1 subsequently recruits the Mediator complex through interactions with MED15. At HSR gene loci, depletion of either MED15 or MED26 leads to reduced Mediator occupancy, indicating that both subunits are required for stable Mediator association at these promoters. In contrast, at nSBs, Mediator accumulation is strictly dependent on MED15, with knockdown of other Mediator subunits (e.g., MED12,^43^ MED25, and MED26) showing relatively little effect on Mediator localization. Notably, while phosphorylation of the HSF1 IDR is essential for HSATIII transcription and nSB formation, its contribution to transcription at HSR gene loci is comparatively modest. Hsf1 forms inducible transcriptional clusters with the Mediator and Pol II to promote the transcription of heat-shock-related genes in *yeast*.^26^ In this context, our findings (summarized in Figure 3) suggest that nSBs (human-specific HSF1 foci) share similarities with yeast Hsf1 condensates in that transcriptional activation in both systems depends on the condensate-forming ability of HSF1, whereas the condensate-forming ability of HSF1 is not essential for the transcription of HSR genes in human cells. Consistently, MED15 may facilitate the formation of HSF1-driven foci (Figures S5G, S5H, and S5I). Despite these differences, Pol II recruitment during heat stress depends on MED26 at both HSR genes and nSBs. Notably, at nSBs, our data suggest that Pol II is recruited to the Mediator in a MED26-dependent manner, possibly revealing a distinct mechanism in which MED26 allosterically promotes the Mediator-Pol II interaction at HSATIII genomic loci. Previous studies have shown that the association of MED26 with the Mediator complex is inhibited by the MED13 IDR, a component of the kinase module, and that dissociation of this module permits MED26 binding; MED26 association is thought to induce a conformational change in the Mediator that enhances its interaction with Pol II.^27^^,^^30^ An important question for future studies is whether the kinase module, including CDK8, functions as a molecular switch regulating MED26 incorporation into Mediator complexes at nSBs. While previous studies have suggested that the kinase module acts as a switch to affect Mediator function,^53^ its role in nSBs has not been determined. In addition, Hsf1 may independently recruit multiple Mediator-related complexes, including complete core Mediator, the MED2-MED3-MED15 tail triad, and the CKM to heat shock-responsive genes of yeast.^54^ This suggests that the Mediator does not function as a single monolithic complex, but rather as a group of dynamically separable complexes that act in a context-dependent manner. Accordingly, it is possible that distinct Mediator complexes, at least those containing MED26, function at HSATIII and HSR gene regions in our study.

Previous studies have implicated MED12 in HSF1 condensate formation at HSP gene promoters.^43^ Our analyses of endogenous nSBs in living human cells revealed that MED15 is more important than MED12 in the formation of nSBs (Figures 5A and S4K). Although MED15 has been proposed to interact with Hsf1 in yeast, human MED15 and HSF1 share limited structural and sequence similarity with their yeast counterparts. Despite this divergence, our results demonstrate that Mediator recruitment via MED15 is conserved in human cells. Importantly, while previous yeast studies did not establish whether MED15 directly interacts with Hsf1,^38^ our *in vitro* and cell-based analyses demonstrate that, as in yeast, MED15 plays a critical role in the heat-stress response in human cells (Figure 5). Furthermore, our findings extend the role of the Mediator beyond HSR gene regulation to transcriptional control within nSBs. Although MED12 promotes HSF1 condensate formation on HSE arrays,^43^ our data raise the possibility that MED15 also contributes to HSF1 condensate dynamics (Figures S5G, S5H, and S5I), consistent with recent reports showing that the Mediator facilitates HSF1 promoter binding.^55^ Elucidating the molecular mechanism by which MED15 engages HSF1 will be an important subject for future studies.

Phosphorylation of HSF1 is a critical determinant of its transcriptional activity and plays an important role in the formation of HSF1 condensates at HSP gene loci.^10^ Our study reveals that phosphorylation of the RD-TAD region of HSF1 has distinct functional effects at HSR genes versus nSBs. Stable expression of a non-phosphorylatable HSF1 modestly reduced HSP gene expression (Figure 3G) but dramatically impaired HSATIII transcription and nSB formation (Figures 3E, 3G, 3H, and 3I). These observations indicate that HSF1 phosphorylation is particularly critical for foci-driven transcriptional programs at nSBs, whereas HSR gene transcription is more tolerant of reduced HSF1 phosphorylation.

Finally, we examined the behavior of the SEC component AFF4 during heat stress. The SEC interacts with the Mediator complex and is preferentially recruited by the NTD of MED26.^35^^,^^56^ We found that AFF4 colocalizes with nSBs upon heat stress (Figure S1F). Notably, depletion of MED26 did not abolish AFF4 colocalization with HSF1 at nSBs (Figure 4G), thereby suggesting that SEC recruitment to nSBs occurs independently of MED26. Deletion of the HR-A/B domain of HSF1, however, reduced AFF4 colocalization with LacI-HSF1 foci (Figure S2G), consistent with the established role of this domain in HSF1 condensate formation.^10^ Together, these findings suggest that SEC recruitment to nSBs is closely linked to HSF1-driven condensate formation. Defining the functional contribution of SEC within nSBs represents an important avenue for future investigation.

### Limitations of the study

Although our results show that MED15 contributes to Mediator recruitment to nSBs and HSR genes in human cells, we were unable to define a direct interaction between HSF1 and MED15. In particular, an *in vitro* binding assay using recombinant MED15 and HSF1 proteins purified from Sf9 cells revealed no detectable direct interaction. In contrast, HSF1 purified from HCT116 cells bound to recombinant MED15, and this interaction was enhanced upon heat stress. These findings suggest that direct binding between HSF1 and MED15 requires heat stress-induced phosphorylation of HSF1 and/or HSF1 trimerization. However, we cannot exclude the possibility that MED15 associates with HSF1 indirectly through an intermediary transcriptional co-regulator that directly binds to HSF1 or that MED15 acts as one of several docking sites within the Mediator complex. Consequently, the precise molecular mechanism by which HSF1 recruits the Mediator to HSR gene loci and nSBs remains unclear. Additionally, our data suggest that the SEC is recruited to HSF1 independently of MED26. Elucidating these mechanisms will be a focus of future studies.

Several caveats should be considered when interpreting the results of ChIP-seq analyses shown in Figure 6. First, the timing of the analysis of different genes following knockdown or induced expression may influence the observed results. Second, ChIP measurements may be affected by antibody quality or differentially exposed epitopes. Finally, the occupancy of the Mediator complex analyzed by ChIP may be minimized, which means that the absence of a detectable signal does not always indicate weak or absent binding. It should also be noted that the overexpression experiments conducted in this study may not fully reflect physiological conditions, since proteins expressed beyond their endogenous levels can exhibit non-native behaviors. In addition, the human cell lines used in this study were derived from both male and female donors. However, biological sex was not examined as an experimental variable, and potential sex-dependent effects on the HSR, HSF1-Mediator interactions, or nSB formation remain to be determined.

## Resource availability

### Lead contact

Requests for further information and resources and reagents should be directed to and will be fulfilled by the lead contact, Hidehisa Takahashi (hide0213@yokohama-cu.ac.jp).

### Materials availability

All unique reagents generated in this study, including genetically modified cell lines and plasmid constructs, are available from the lead contact upon reasonable request and with a completed Materials Transfer Agreement.

### Data and code availability

Original data are available from the lead contact upon request. ChIP-seq and RNA-seq data are deposited in GEO under accession number GEO: GSE318822. The IP-MS proteomics data have been deposited to the ProteomeXchange Consortium via the jPOST repository under accession number ProteomeXchange: PXD073807. The TurboID-MS proteomics data have been deposited under accession number ProteomeXchange: PXD073817. This paper does not report original code. Any additional information required to reanalyze the data reported in this paper is available from the lead contact upon reasonable request.

## Acknowledgments

We thank Miho Uchiumi, Misa Kobayashi, and Kaori Shirouzu for assisting with the manuscript preparation. We also thank Michelle Kahmeyer-Gabbe, PhD, from Edanz (https://jp.edanz.com/ac) for editing a draft of this manuscript. This research was supported by the following: AMED-PRIME grant 25gm6910006h0003 to H.T.; JSPS 10.13039/501100001691KAKENHI grants 16H06279 and 221S0002 (PAGS); 10.13039/501100001691KAKENHI grants 2020K15718 to H.S., 20H03182 to Y.Y., and 17K19578, 18H02378, 19K22401, 21H05159, 21H02405, 21K19356, 24K01958,and 25K22456 to H.T.; funding by 10.13039/100007449Takeda Science Foundation, Takamatsu Cancer Research Fund, Chugai Foundation for Innovative Drug Discovery Science, Leukemia Research Fund, The 10.13039/501100004398Mitsubishi Foundation, The 10.13039/100008608Sumitomo Foundation, The Ichiro Kanehara Foundation, Friends of Leukemia Research Fund, Ono Cancer Research Fund, 10.13039/501100007533Kobayashi Foundation for Cancer Research, MSD Life Science Foundation, The 10.13039/100007428Naito Foundation, The Tokyo Biochemical Research Foundation, The Uehara Memorial Foundation, Yokohama Foundation for Advancement of Medical Science, 10.13039/501100008884Nakatani Foundation, Daiichi Sankyo Foundation of Life Science, and Research Foundation for Opto-Science and Technology to H.T.; and funding by 10.13039/501100013236MSD Life Science Foundation, Takeda Science Foundation, and SENSHIN Medical Research Foundation to H.S.

## Author contributions

Conceptualization, K.L., K.F., H.S., and H.T.; methodology, K.L., K.F., H.S., S.S., N.Y., Y.I., K.H., and H.T.; validation, K.L.; formal analysis, R.A.; investigation, K.L., K.F., H.S., and H.T.; resources, H.T.; writing – original draft, K.L. and H.T.; supervision, H.T.; funding acquisition, H.S. and H.T.

## Declaration of interests

The authors declare no competing interests.

## Declaration of generative AI and AI-assisted technologies in the writing process

The authors did not use generative AI or AI-assisted technologies in the preparation of this manuscript.

## STAR★Methods

### Key resources table

**Table:** 

REAGENT or RESOURCE	SOURCE	IDENTIFIER
**Antibodies**		

Anti-HSF1	Santa Cruz Biotechnology	Cat# sc-13516; RRID: AB_627751
Anti-MED26 (D4B1X)	Cell Signaling Technology	Cat# 14950S; RRID: AB_2798656
Anti-MED1	Bethyl Laboratories	Cat# A300-793A; RRID: AB_577241
Anti-TRAP220/MED1 (ChIP)	Abcam	Cat# Ab64965; RRID: AB_1142301
Anti-MED12	Bethyl Laboratories	Cat# A300-774A; RRID: AB_669756
Anti-MED8	Santa Cruz Biotechnology	Cat# sc-103619
Anti-MED17	Cell Signaling Technology	Cat# 64733
Anti-MED25	Abcam Antibodies	Cat# Ab221741
Anti-CDK8	Thermo Fisher	Cat# A302-500A; RRID: AB_1966093
Anti-AFF4	Santa Cruz Biotechnology	Cat# sc-390310; RRID: AB_2924396
Anti-Pol II CTD	MBL	Cat# MABI0601; RRID: AB_2728735
Anti-Coilin	Abcam	Cat# ab11822; RRID: AB_2081428
Anti-KIAA1429	Atlas	Cat# HPA031530; RRID: AB_10600818
Anti-ICE1	Bethyl Laboratories	Cat# A304-276A; RRID: AB_2620472
Anti-HA	Roche	Cat# 11867423001; RRID: AB_390918
Anti-V5	Invitrogen	Cat# R960-25; RRID: AB_2556564
Anti-FLAG-M2-HRP	MilliporeSigma	Cat# A8592; RRID: AB_439702
Anti-V5-HRP	Thermo Fisher	Cat# 46–0708; RRID: AB_2556565
Goat anti-rat IgG Alexa Fluor 555	Invitrogen	Cat# A21434; RRID: AB_2535855
Goat anti-rat IgG Alexa Fluor 647	Invitrogen	Cat# A21247; RRID: AB_141778
Goat anti-rabbit IgG Alexa Fluor 488	Invitrogen	Cat# A11034; RRID: AB_2576217
Goat anti-rabbit IgG Alexa Fluor 647	Invitrogen	Cat# A21245; RRID: AB_2535813
Goat anti-mouse IgG Alexa Fluor 555	Invitrogen	Cat# A21127; RRID: AB_2535769
Goat anti-mouse IgG Alexa Fluor 647	Invitrogen	Cat# A21236; RRID: AB_2535805
Donkey anti-goat IgG Alexa Fluor 555	Invitrogen	Cat# A21432; RRID: AB_2535853
Goat anti-rat IgG HRP	Cell Signaling Technology	Cat# 7077S; RRID: AB_10694715
Goat anti-rabbit IgG HRP	Cell Signaling Technology	Cat# 7074S; RRID: AB_2099233
Horse anti-mouse IgG HRP	Cell Signaling Technology	Cat# 7076S; RRID: AB_330924

**Bacterial and virus strains**		

DH10bac	Thermo Fisher	10361012
DH5α	Takara Bio	9057
Recombinant baculovirus expressing FLAG-MED15	This study	N/A

**Chemicals, peptides, and recombinant proteins**		

Actinomycin D	Sigma-Aldrich	A1410
Formaldehyde	Wako	063–05991
DMSO (Dimethyl Sulfoxide)	Nacalai tesque	13445–74
dTAGv-1	Tocris Bioscience	6914

**Critical commercial assays**		

iTaq Universal SYBR Green One-Step Kit	Bio-Rad	1725151
THUNDERBIRD Next SYBR qPCR Mix	Toyobo	QPX-201
Immobilon Forte Western HRP Substrate	Millipore	WBLUF0500

**Deposited data**		

MED26-IP mass spectrometry data	This study	JPOST: JPST004360/PXD073807
TurboID-HSF1 SILAC mass spectrometry data	This study	JPOST: JPST004361/PXD073817
HSF1-Mutant RNA-seq data	This study	GEO: GSE318822
dTAG-MED26 RNA-seq data	This study	GEO: GSE318822
HSF1-Mutant MED26-ChIP-seq data	This study	GEO: GSE318822
dTAG-MED15 MED1-ChIP-seq data	This study	GEO: GSE318822
dTAG-MED15 MED26-ChIP-seq data	This study	GEO: GSE318822
dTAG-MED26 MED1-ChIP-seq data	This study	GEO: GSE318822
dTAG-MED26 Pol II-ChIP-seq data	This study	GEO: GSE318822

**Experimental models: Cell lines**		

HeLa human cervical adenocarcinoma cells; female origin	ATCC	CCL-2
HCT116 human colorectal carcinoma cells; male origin	ATCC	CCL-247
U2OS human osteosarcoma cells; female origin	ATCC	HTB-96
HEK293T human embryonic kidney cells; female origin	ATCC	CRL-3216
Lenti-X 293T cells; HEK293T-derived, female origin	Takara Bio	Cat. no. 632180
Sf9 insect cells; female origin	Thermo Fisher Scientific	Cat# 11496015; RRID: CVCL_0549

**Oligonucleotides**		

HSATIII RNA-FISH probe (3′-Cy3 labeled)		
5′-ATT CCA TTC CAT TCC ATT CC-3′ Cy3Sp	In this study	N/A
Primers used for qPCR analysis		
HSATIII lncRNA	In this study	N/A
5′-TATGAATTCAATCAACCCGAGTGCAATCGAA-3′ (forward)		
5′-TATGGATCCTTCCATTCCATTGCTGTACTCG-3′ (reverse)		
HSPA1A	In this study	N/A
5′-TGCTGGACAAGTGTCAAGAGGTCA-3′ (forward)		
5′-TCCTCTTGTGCTCAAACTCGTCCT-3′ (reverse)		
HSPA1B	In this study	N/A
5′-GCAAGGCCAACAAGATCACC-3′ (forward)		
5′-TCAAACTCGTCCTTCTCGGC-3′ (reverse)		
GAPDH	In this study	N/A
5′-TCGACAGTCAGCCGCATCTTCTTT-3′ (forward)		
5′-GCCCAATACGACCAAATCCGTTGA-3′ (reverse)		
HSPA1A promoter	In this study	N/A
5′- TCTGATTGGTCCAAGGAAGGCTG-3′ (forward)		
5′- TTTCCCTTCTGAGCCAATCACCGA-3′ (reverse)		
HSPA1B promoter	In this study	N/A
5′-GAGGGTCCGCTTCGTCTTTC-3′(forward)		
5′-GAACGCCGGAAACTCAACAC-3′(reverse)		

**Recombinant DNA**		

LacI-HSF1 constructs	In this study	N/A
CRY2-mCherry-HSF1	In this study	N/A
EGFP-MED26 constructs	In this study	N/A
EGFP-MED15 constructs	In this study	N/A

**Software and algorithms**		

ImageJ/Fiji	Fiji: an open-source platform for biological- image analysis	https://fiji.sc/
Fiji/ImageJ	Schindelin et al.	https://fiji.sc
Progenesis QI for Proteomics	Waters	https://www.nonlinear.com
MACS2	Zhang et al.	https://github.com/macs3-project/MACS

### Experimental model and study participant details

#### Cell line generation

HeLa human cervical adenocarcinoma cells (CCL-2; American Type Culture Collection [ATCC], Manassas, VA, USA) were derived from a 31-year-old female donor. HCT116 human colorectal carcinoma cells (CCL-247; ATCC) were derived from a 48-year-old male donor. U2OS human osteosarcoma cells (HTB-96; ATCC) were derived from a 15-year-old female donor. HEK293T human embryonic kidney cells (CRL-3216; ATCC) were derived from fetal tissue of female origin. Lenti-X 293T cells (632180; Takara Bio, Shiga, Japan), a HEK293T-derived lentiviral packaging cell line of female fetal origin, were used for lentivirus production.

HeLa, HCT116, U2OS, HEK293T, and Lenti-X 293T cells were cultured in Dulbecco’s modified Eagle’s medium (DMEM, 08458-45; Nacalai Tesque, Kyoto, Japan) supplemented with 10% fetal bovine serum and 1% penicillin–streptomycin at 37°C in a humidified atmosphere containing 5% CO2. For heat-shock experiments, cells were incubated at 43°C for 1 h.

Sf9 insect cells (11496015; Thermo Fisher Scientific, Waltham, MA, USA) were derived from pupal ovarian tissue of female Spodoptera frugiperda and were used for baculovirus-mediated recombinant protein production. Sf9 insect cells (11496015; Gibco, Thermo Fisher Scientific) were maintained in Sf-900 II serum-free medium (10902096; Gibco, Thermo Fisher Scientific) supplemented with 5% fetal bovine serum (FBS) at 27°C without supplemental CO_2_.

Genetically modified HCT116-derived cell lines used in this study included dTAG-HSF1, dTAG-MED12, dTAG-MED15, dTAG-MED25, and dTAG-MED26 cells. Additional derivative cell lines expressed TurboID-HSF1, doxycycline-inducible HSF1-WT or HSF1-S>A, MED26-WT or MED26 mutants, and MED15-WT or MED15 mutants, as described below. The relevant genetic modifications were introduced using CRISPR-Cas9-mediated genome editing or lentiviral transduction.

All cell lines were routinely tested for mycoplasma contamination and were confirmed to be negative. The cell lines were obtained from the indicated commercial sources but were not independently authenticated after purchase. No animals, human participants, or primary human samples were used in this study; therefore, institutional animal or human research approval was not applicable.

#### Bacterial and virus strains

Escherichia coli DH5α cells were used for plasmid propagation. Transformed cells were cultured in LB medium supplemented with the appropriate selective antibiotics at 37°C. Escherichia coli DH10Bac competent cells (10361012; Thermo Fisher Scientific) were used to generate recombinant bacmid DNA using the Bac-to-Bac baculovirus expression system according to the manufacturer’s instructions. Recombinant baculoviruses encoding FLAG-tagged MED15 were generated by transfecting the purified bacmid DNA into Sf9 cells and were used exclusively for recombinant protein production.

### Method details

#### DNA constructs and mutagenesis

A cDNA clone of *HSF1* (MHS6278-202828505; DHA (Horizon Discovery); Cambridge, UK) was PCR-amplified and purified using the QIAquick PCR Purification Kit (28106; Qiagen, Hilden, Germany). *MED15* cDNA was generated from total RNA of HEK293T cells using the cDNA Library Kit (1708897; Bio-Rad, Hercules, CA, USA), amplified by PCR, and cloned into vector pEGFP.

Plasmids used for transient expression, including LacI-GFP-NLS-HSF1, LacI-RFP-NLS-HSF1-Mutants, CRY2-mCherry-HSF1, GFP-MED15, and GFP-MED26, were PCR-amplified and purified using the QIAquick PCR Purification Kit. Some plasmids were generated using In-Fusion cloning methods, while point mutations were introduced via PCR mutagenesis. The resulting constructs were transformed into *Escherichia coli* DH5α, and transformants were selected on LB agar plates supplemented with the appropriate antibiotic and incubated at 37°C for >15 h. Single colonies were picked and cultured in 3 mL of LB medium containing antibiotics at 37°C for >15 h. Plasmid DNA was purified using the NucleoSpin Plasmid EasyPure Kit (740727; Takara Bio).

#### Lentiviral-mediated generation of stable, inducible cell lines

Stable cell lines expressing HSF1 and MED15 mutants were generated using a lentiviral system. Briefly, the TRIPZ lentiviral doxycycline (Dox)-inducible plasmid (Open Biosystems, Huntsville, AL, USA) was transfected into Lenti-X 293T cells and the culture medium containing viral particles was collected and used to replace part of the culture medium of cells with stable dTAG-mediated knockdown of HSF1 or MED15. On day 3 post-infection, blasticidin (final concentration: 10 ng/μL) was added for selection.

#### Plasmid transfection

Cells were seeded in culture plates and transfected with plasmids the next day using Lipofectamine 2000 (11668019; Invitrogen, Carlsbad, CA, USA) and Opti-MEM I Reduced Serum Medium (31985070; Gibco, Grand Island, NY, USA). Cells were subjected to experimental treatments at 24 h post-transfection.

#### Production of recombinant proteins

Recombinant FLAG-tagged MED15 was expressed in Sf9 cells (11496015; Thermo Fisher Scientific) using the Bac-to-Bac baculovirus expression system (Thermo Fisher Scientific). The FLAG-MED15 coding sequence was subcloned into the pFastBac vector and transformed into *E. coli* DH10Bac (10361012; Thermo Fisher Scientific) to generate recombinant bacmid DNA. Purified bacmid DNA was transfected into Sf9 cells to produce recombinant baculovirus, and infected Sf9 cells were used for protein expression. FLAG-MED15 was purified using anti-FLAG M2 Affinity Gel (A2220; Sigma-Aldrich, St. Louis, MO, USA) and eluted with 3X FLAG Peptide (F4799; Sigma-Aldrich) at a concentration of 250 μg/mL.

#### FISH and IF

For IF, HCT116 or U2OS cells were cultured on coverslips, fixed with 4% paraformaldehyde (PFA) for 10 min, permeabilized with 0.3% Triton X-100 for 15 min, and blocked for 60 min in blocking buffer (phosphate-buffered saline [PBS] containing 10% bovine serum albumin and 2% horse serum). Cells were then incubated with the following primary antibodies diluted in blocking buffer for 60 min: anti-HSF1 (1:100, sc-13516; Santa Cruz Biotechnology, Santa Cruz, CA, USA), anti-MED26 (D4B1X, 1:200, 14950S; Cell Signaling Technology, Danvers, MA, USA), anti-AFF4 (1:100, sc-390310; Santa Cruz Biotechnology), anti-Pol II (1:1000, MABI0601; MBL, Tokyo, Japan), anti-coilin (1:2000, ab11822; Abcam, Cambridge, UK), anti-v5 (1:500, 46-0705; Invitrogen), anti-KIAA1429 (1:100, HPA031530; Atlas Antibodies, Bromma, Sweden), anti-ICE1 (1:300, A304-276A; Bethyl Laboratories, Montgomery, TX, USA), anti-MED8 (1:100, sc-103619; Santa Cruz Biotechnology), anti-MED12 (1:500, A300-774A; Bethyl Laboratories), anti-CDK8 (1:300, sc-1521; Santa Cruz Biotechnology), anti-MED17 (1:200, 64733; Cell Signaling Technology), and anti-MED1 (1:200, A300-793A, Bethyl Laboratories). Cells were washed with PBS and incubated for 45 min with the following secondary antibodies diluted 1:2000 in PBS: goat anti-rabbit IgG Alexa Fluor 488 (A11034; Invitrogen), goat anti-rabbit IgG Alexa Fluor 647 (A21245; Life Technologies, Carlsbad, CA, USA), goat anti-mouse IgG Alexa Fluor 647 (A21236; Invitrogen), and goat anti-rat IgG Alexa Fluor 555 (A21434; Invitrogen). Nuclei were counterstained with 1 mM DAPI (11034-5; Nacalai Tesque). Coverslips were mounted onto glass slides using ProLong Glass Antifade Reagent (Invitrogen), and images were acquired using a Zeiss LSM 700 laser scanning microscope (Carl Zeiss, Oberkochen, Germany).

For RNA-FISH, HCT116 cells were cultured on coverslips as described for IF, treated, and immediately fixed at room temperature for 15 min in fixation buffer (4% PFA, 0.5% Triton X-100, 40×ribonucleoside–vanadyl complex, in PBS). Cells were then dehydrated in 75% ethanol for 5 min and permeabilized for 20 min in permeabilization buffer (0.5% Triton X-100, 40× ribonucleoside–vanadyl complex, in PBS). After permeabilization, cells were washed with 2× saline sodium citrate (SSC) buffer containing 0.05% Tween-20 for 5 min, followed by prehybridization in 50% formamide in 2× SSC. Probes were dissolved in formamide and denatured at 85°C for 5 min, then diluted in an equal volume of hybridization buffer (4× SSC, 20% sodium dextran sulfate, 40× ribonucleoside–vanadyl complex, in RNase-free water). A 10-μL volume of diluted probe was applied to each slide, coverslips were mounted, and edges were sealed with paper bond. Hybridization was carried out overnight in a humid chamber at 37°C with light shielding. The following day, coverslips were washed twice for 5 min each with 50% formamide in 2× SSC prewarmed to 37°C, followed by two washes in 2× SSC. Subsequently, IF staining was performed to detect target proteins.

#### RNA purification and DNase removal of genomic DNA

Cells cultured in 35-mm dishes were harvested and lysed in 500 μL of Sepasol (Nacalai Tesque) at room temperature for 5 min to disrupt nuclear protein complexes. Subsequently, 100 μL of chloroform was added and the tube was shaken vigorously for 15 s, incubated at room temperature for 3 min, and centrifuged at 13,000 × *g* for 15 min. The aqueous phase was transferred to a new tube and mixed with an equal volume of isopropanol. After incubation at room temperature for 10 min, the mixture was centrifuged, the supernatant was discarded, and the pellet was washed with 500 μL of 75% ethanol, vortexed, and centrifuged again. The RNA pellet was then dissolved in RNase-free water. To remove genomic DNA, the RNA samples were treated with Turbo DNase (1 μL; AM2238, Invitrogen) in 1× Turbo DNase buffer (10× stock, 4022G; Invitrogen) at 37°C for 30 min, followed by a second round of RNA purification using Sepasol.

#### qRT-PCR and quantitative PCR (qPCR)

Purified RNA was used as template for qRT-PCR with the iTaq Universal SYBR Green One-Step Kit (1725151; Bio-Rad), in accordance with the manufacturer’s protocol. Threshold cycle (Ct) values were determined using the CFX Connect Real-Time PCR Detection System (Bio-Rad), and relative expression levels were calculated using the comparative Ct method. qPCR was performed in THUNDERBIRD Next SYBR mix (QPX-201; Toyobo, Osaka, Japan), following the manufacturer’s instructions.

#### Western blotting

After removing the culture medium and washing once with PBS, cells cultured in 35-mm dishes were harvested and pelleted by centrifugation. The supernatant was discarded, and the cell pellet was resuspended in RIPA buffer (50 mM Tris-HCl pH 8.0, 150 mM NaCl, 1% Triton X-100, 0.5% sodium deoxycholate [Doc], 0.1% sodium dodecyl sulfate [SDS]) supplemented with protease inhibitors. The lysates were then sonicated using a Bioruptor sonicator (Diagenode, Denville, NJ, USA) for 10 min on the high setting (30 s on/30 s off), incubated on ice for 10 min, and centrifuged to collect the supernatant as whole-cell extracts. Protein concentrations were determined by the BCA assay, and aliquots corresponding to 10 μg of protein were diluted in 5× SDS sample buffer (0.31 mM Tris-HCl [pH 6.8], 10% SDS, 25% sucrose, 0.025% bromophenol blue, 25% β-mercaptoethanol). Samples were boiled at 95°C for 5 min, loaded onto 10% Mini-PROTEAN TGX gels (Bio-Rad), and separated by SDS-PAGE in running buffer (192 mM glycine, 25 mM Trizmabase, 0.1% SDS) at 20 mA/gel for 50 min using a PowerPac Basic power supply (Bio-Rad). Proteins were then transferred to Immobilon-P polyvinylidene fluoride membranes (IPVH00005; Millipore, Burlington, MA, USA) that had been preactivated in methanol for 5 min. Transfer was performed in transfer buffer (192 mM glycine, 25 mM Tris base, 20% methanol) at 100 V for 1 h under cooled conditions. Membranes were blocked in Blocking One solution (03953-95; Nacalai Tesque) for 1 h and then incubated for 1 h with the following primary antibodies diluted in Tris-buffered saline with 0.05% Tween-20 (TBS-T): anti-HSF1 (1:2000, sc-13516; Santa Cruz Biotechnology) and anti-MED26 (D4B1X, 1:5000, 14950S; Cell Signaling Technology). After three washes with TBS-T, membranes were incubated for 1 h with the following horseradish peroxidase (HRP)-conjugated secondary antibodies diluted in TBS-T: anti-v5-HRP (1:5000, 46-0708; Invitrogen), anti-rabbit IgG-HRP (1:5000, 7074S; Cell Signaling Technology), anti-mouse IgG-HRP (1:5000, 7076S; Cell Signaling Technology), and anti-rat IgG-HRP (1:5000, 7077S; Cell Signaling Technology). Membranes were washed three times with TBS-T, and protein bands were visualized using the Immobilon Forte Western HRP Substrate (WBLUF0500; Millipore) with an Amersham ImageQuant 800 system (Cytiva, Marlborough, MA, USA).

#### Protein IP–MS

Protein complexes were purified from nuclear extracts of cell lines stably expressing FLAG-tagged proteins. Nuclear extracts and S100 fractions were prepared from parental HeLa cells following the method described by Dignam et al.^57^ Extract fractions were incubated with 40 μL of anti-FLAG M2 Affinity Gel (A2220; Sigma-Aldrich) for 1 h at 4 °C. The beads were washed five times with IP wash buffer (50 mM Hepes-KOH, pH 7.9, 300 mM NaCl, 1 mM EDTA, 10% glycerol, 0.1% Triton X-100), and bound proteins were eluted with 60 μL of FLAG peptide (0.5 μg/μL) for 20 min at room temperature. The eluates were checked by western blotting, and immunoprecipitates were precipitated by adding four volumes of ice-cold 100% acetone and incubating overnight at −80°C. The next day, the acetone was removed, and the pellets were washed once with 200 μL of ice-cold 80% acetone. Protein pellets were resuspended in 20 μL of 8 M urea/50 mM NH_4_HCO_3_, reduced with 2 μL of 100 mM dithiothreitol (DTT) for 30 min at 37°C, and alkylated with 2 μL of 250 mM iodoacetamide for 15 min at room temperature. Proteins were digested with 0.5 μg of trypsin (in 60 μL of 50 mM N NH_4_HCO_3_) at 37°C for 17 h. Digestion was terminated by adding 1.5 μL of 20% trifluoroacetic acid (TFA).

Peptides were desalted and concentrated using StageTips packed with two disks of Empore styrene-divinylbenzene material (5010-30045; GL Sciences, Tokyo, Japan) and three disks of Empore C18 octadecyl material (2215; 3M) and placed into low-retention pipette tips (D200; Gilson, Middleton, WI, USA). StageTips were pre-equilibrated with 100 μL of buffer B60 (0.1% TFA, 60% acetonitrile [ACN]) and 100 μL of buffer A (0.1% TFA, 2% ACN) by centrifugation. Samples were loaded, washed four times with buffer A, and peptides were eluted sequentially with 50 μL of buffer B30 (0.1% TFA, 30% ACN) and 50 μL of buffer B60 by centrifugation. The eluted peptides were dried and concentrated using a SpeedVac (Thermo Fisher) prior to the MS analysis. Peptides were identified using Mascot Server, and a quantitative analysis was conducted using Progenesis QI for proteomics. Protein enrichment scores (SCORE) were calculated as follows:

$$SCORE=\frac{\left(Normalized\;abundance\;of\;Flag-tagged\;cell\;samples\right)+100}{\left(Normalized\;abundance\;of\;parental\;HeLa\;cell\;samples\right)+100}$$

#### SILAC

Cell lines with stable TurboID-HSF1 expression were checked for biotinylation using western blotting and IF. Cells were then cultured for 1 week in three SILAC media conditions: Light (DMEM SILAC 88364; Thermo Fisher Scientific, with dialyzed FBS, l-lysine hydrochloride, l-arginine hydrochloride); Medium (same DMEM with dialyzed FBS, l-lysine-^13^C6 hydrochloride, l-arginine-^13^C6 hydrochloride), and High (same DMEM with dialyzed FBS, l-lysine-^13^C6, ^15^N2 hydrochloride, l-arginine-^13^C6, ^15^N2 hydrochloride) to label the amino acids. Cells were then seeded in 6-cm plates, and Dox and dTAG were added the next day to deplete intrinsic HSF1 and activate the expression of TurboID-HSF1. At 24 h after adding Dox and dTAG, 100 nM biotin was added to the medium and incubated for 1 h. For heat-shock conditions, cells were placed into a 43°C incubator immediately after adding biotin. Cells were washed three times with PBS, harvested using a cell scraper, and resuspended in EB300 buffer (300 mM NaCl, 1% NP-40, 1 mM EDTA, 1 mM MgCl_2_, 50 mM Tris-HCl pH 7.5, protease inhibitor). The lysates were sonicated for 10 min and incubated on ice for another 10 min. The supernatant was collected and the protein concentrations were determined by the BCA assay. Equal amounts of Light, Medium, and High samples were combined in a single tube, after which 30 μL of Streptavidin MyOne T1 beads was added and incubated at 4°C for 3 h. The beads were washed twice with EB300 buffer and twice with 0.5 M NaCl RIPA buffer and then eluted for MS analyses. This experiment was performed in triplicate (*n* = 3).

#### ChIP

HCT116 cells (4.5 × 10^6^ cells from one 10-cm dish, 2 days after seeding) were used for ChIP assays, which were performed as described previously.^44^ Following the indicated treatments, cells were cross-linked with disuccinimidyl glutarate (in PBS) for 30 min at room temperature, washed once with PBS, and then cross-linked with 1% formaldehyde for 15 min at room temperature. Cross-linking was terminated by adding 1.25 M glycine (final concentration, 0.125 M) for 5 min. Cells were washed once with PBS, harvested in 4 mL of ice-cold PBS using a cell scraper, washed twice with PBS, and resuspended in NP-40 lysis buffer (10 mM HEPES-KOH, pH 7.8, 10 mM KCl, 0.1 mM EDTA, 0.5% NP-40). After incubation on ice for 5 min, cells were resuspended and lysed in ChIP lysis buffer (25 mM Tris-HCl, pH 7.5, 800 mM NaCl, 5 mM EDTA, 0.5% Doc, 0.1% SDS, 1% Triton X-100). Chromatin was sheared using a Bioruptor sonicator (30 cycles × 30 s on/30 s off, at maximum power) to generate DNA fragments of 150–400 bp. Lysates were centrifuged at 13,000 × *g* for 10 min, and supernatants were collected. For input DNA, 21 μL of the clarified lysate was used. After diluting the 210 μL lysate volume with 700 μL of dilution buffer (25 mM Tris-HCl pH 7.5, 5 mM EDTA, 0.1% SDS, 1% Triton X-100), 2–4 μg of the following antibodies were added, followed by rotation overnight at 4°C: anti-MED26 (D4B1X, 14950S; Cell Signaling Technology), anti-MED1 (ab64965; Abcam), and anti-Pol II CTD (MABI0601; MBL).

Protein G magnetic beads were prewashed once with bead equilibration buffer (25 mM Tris-HCl, pH 7.5, 5 mM EDTA, 0.1% SDS, 1% Triton X-100, 184 mM NaCl, 0.1% Doc), added to the lysates, and incubated at 4°C for 3 h. Beads were then washed with a series of buffers, each performed for 5 min at 4°C with rotation, followed by removal of the supernatant: once each with 1 mL of low-salt wash buffer (0.1% SDS, 1% Triton X-100, 2 mM EDTA, 150 mM NaCl, 20 mM Tris–HCl pH 8.0), high-salt wash buffer (0.1% SDS, 1% Triton X-100, 2 mM EDTA, 500 mM NaCl, 20 mM Tris–HCl pH 8.0), and LiCl wash buffer (0.25 M LiCl, 1% NP-40, 1% Doc, 1 mM EDTA, 10 mM Tris–HCl pH 8.0); and twice with TE buffer (1 mM EDTA, 10 mM Tris–HCl pH 8.0). Chromatin was eluted from the beads in 200 μL of ChIP elution buffer (1% SDS, 100 mM NaHCO_3_) at 65°C for ≥20 min. NaCl was added to a final concentration of 0.2 M, and samples were incubated at 65°C overnight to reverse cross-links. Immunoprecipitated and input DNA were treated with RNase A at 37°C for 30 min, followed by proteinase K digestion at 55°C for ≥2 h. DNA was purified using the MinElute PCR Purification Kit (Qiagen).

#### Luciferase reporter assays

HEK293T cells were seeded into 96-well plates and transfected with plasmids (5 ng of pGL4.31 and pBIND-HSF1-mutant) 24 h later. Firefly and *Renilla* luciferase activities were measured using the Dual-Luciferase Reporter Assay System (Promega, Madison, WI, USA), in accordance with the manufacturer’s protocol. Each experiment was performed in triplicate.

#### *In vitro* pulldown assay

FLAG-tagged MED15 protein was expressed in Sf9 cells using a baculovirus expression system and purified for use in pulldown assays. A FLAG-MED15 expression construct was generated in the pFastBac vector, and recombinant baculovirus was used to infect Sf9 cells. Robust expression of FLAG-MED15 was confirmed by western blotting. Infected Sf9 cells were cultured in 10-cm dishes, harvested, and FLAG-MED15 protein was purified using anti-FLAG affinity beads.

Briefly, cells were washed twice with PBS and lysed in 500 μL of lysis buffer (0.5% TritonX-100, 150 mM NaCl, 40 mM Hepes pH 7.9, 1.5 mM MgCl_2_, 0.5 mM DTT, protein inhibitor) on ice for 10 min with vortexing. Following centrifugation, the supernatant was collected as lysate and incubated with 100 μL of anti-FLAG M2 Affinity Gel (A2220; Sigma-Aldrich) for 1 h at 4°C. Beads were washed five times with IP wash buffer (50 mM Hepes-KOH, pH 7.9, 300 mM NaCl, 1 mM EDTA, 10% glycerol, 0.1% Triton X-100), and bound proteins were eluted twice with 100 μL of FLAG peptide (500 μg/mL) for 20 min at room temperature. Purified proteins were confirmed by western blotting.

For pulldown assays, HCT116 cells stably expressing HSF1-WT or HSF1 S>A under endogenous HSF1 knockdown were lysed as described above. Protein G magnetic beads (30 μL) were prewashed once with PBS and incubated with 2 μg of v5-antibody (R96025; Invitrogen) per sample at 4°C for 30 min. After washing twice with PBS, v5-coupled beads were mixed with the HSF1-v5 (WT and S>A mutant) cell lysates and incubated at 4°C for 1 h. Beads were washed three times with IP wash buffer (150 mM NaCl, 1 mM EDTA, 50 mM Hepes-KOH pH 7.9, 10% glycerol, 0.1% Triton X-100), followed by incubation with 10 μL of purified FLAG-MED15 proteins at 37°C for 30 min. After three additional washes with IP wash buffer, bound proteins were eluted twice with v5 peptide at 37°C for 30 min.

### Quantification and statistical analysis

#### Bioinformatic analysis of SILAC-MS data

SILAC-MS data were processed and analyzed using R. Protein intensities from three experimental conditions (L, M, and H, each with three replicates) were extracted for downstream analyses. Zero values were treated as missing, and intensities were log2-transformed. Mean intensities for each condition (L for negative control, M for normal temperature, and H for heat shock) were calculated, and pairwise log2 fold changes were determined. Statistical significance was assessed using Student’s *t*-tests, followed by Benjamini-Hochberg correction for multiple testing. Proteins with adjusted *p*-values <0.05 were considered significant. Volcano plots were generated using ggplot2. Mediator complex subunits were highlighted in the analysis.

For the STRING network analysis summarized in Figure 1E, we applied an additional filtering criterion based on replicate-level comparisons. Proteins were retained if their intensities in at least two out of three replicates were higher in both H/L and M/L conditions. Proteins meeting this criterion and showing statistical significance (adjusted *p*-value <0.05) were selected for downstream analyses. The resulting gene list was used for a protein–protein interaction analysis using STRING (version 12.0). High-confidence interactions were selected, and k-means clustering was applied to group proteins into clusters; only clustered nodes were visualized.

#### Bioinformatic analysis of RNA-seq data

Total RNA was purified using the Sepasol method. RNA-seq libraries were produced using the NEBNext Ultra II Directional RNA Library Prep Kit for Illumina (rRNA Depletion Kit v2, E7400L; New England Biolabs, Ipswich, MA, USA). Raw paired-end RNA-seq reads were quality-checked using FastQC (v0.12.1) and processed using fastp with the default quality-filtering thresholds. Bases with Phred quality scores below 15 were considered unqualified, and reads in which more than 40% of bases were unqualified were removed, generating clean FASTQ files. Reads were aligned to the human reference genome GRCh38 using STAR (v2.7.8a), with default parameters; the STAR index was generated based on the *Homo sapiens* GRCh38 primary assembly and corresponding Ensembl gene annotation (release 112). Aligned, coordinate-sorted BAM files were indexed using SAMtools (v1.16.1), and genome-wide coverage tracks were generated using bamCoverage with CPM normalization to produce bigWig files for visualization. Transcript and gene expression levels were quantified using RSEM (v1.3.1) based on the STAR-aligned transcriptome BAM files.

#### Bioinformatic analysis of ChIP-seq data

Next-generation sequencing (NGS) libraries were produced using the NEBNext Ultra II DNA Library Prep Kit for Illumina (E7645L; New England Biolabs). Adapter sequences were trimmed and reads were quality-filtered using fastp (v0.23.2). Processed reads were mapped to the human genome GRCh38 using Bowtie2 (v2.4.2, 64-bit) with default parameters. Mapped reads were converted to sorted BAM files and indexed using SAMtools (v1.16.1). Normalized genome coverage tracks were generated using bamCoverage with CPM normalization. Peaks were identified using MACS2 (v2.2.7.1) and annotated, with a motif analysis performed using HOMER. For spike-in normalization, reads were aligned to the *Drosophila melanogaster* (dm6) genome, and normalization factors calculated from *Drosophila*-mapped reads were applied to generate spike-in-normalized bigWig files.

#### Bioinformatic analysis of HSATIII repeat RNA from NGS data

Genomic regions corresponding to HSATIII and HSATII satellite repeats were obtained from the chm13 v2.0 centromeric satellite (cenSTA) v2.1 assembly, and sequences therein were extracted using BEDTools (v2.31.1). A reference dataset for hidden Markov model (HMM)-based analysis using HMMER was generated using SeqKit (v2.10.0) with a sliding window size of 200 bp and step size of 50 bp. Only sequence windows containing one or more of the following motifs were retained: ATTCC, TTCCA, TCCAA, CCAAT, or CAATT. Retained sequences were clustered using CD-HIT-EST (v4.8.1; parameters: -c 0.85 and -n 8), followed by alignment with MAFFT (v7.526) using the options --globalpair, --allowshift, and --maxiterate 1000. HMM profiles were then constructed using HMMER (v3.4) with default parameters. Motif searches were conducted on individual reads using nhmmer, and hits with an E-value <1 × 10^-10^ were considered significant.

#### Composite image generation and quantitative analysis

Nuclei were segmented and cytoplasmic signals were excluded. LacI or HSF1 foci were identified, and their centroid coordinates were determined with a precision of 0.1 pixels. The occupancy of Lac-I foci within each nucleus was calculated, and foci originating from overexpressing cells were excluded using a 5% upper-occupancy threshold. Original images were upscaled fivefold in resolution, and images from each channel were extracted around each LacI centroid and then stacked. Average signal intensity across the stacks was calculated to generate composite images, and signal intensities were visualized using the mpl-inferno color scale. Line plots were generated along an axis passing through each centroid.

## Supplemental information

Document S1. Figures S1–S5

Table S1. Genes classified as heat-shock response or putative heat-shock response genes based on integrated RNA-seq and ChIP-seq analyses, related to Figure 1

Data S1. Source data underlying the quantitative analyses presented in Figures 1–6 and S1–S5
