# Supplementary material for: MED26 facilitates Mediator-Pol II complex assembly and promotes nuclear stress body formation during heat stress
Source: iScience. 2026 Sep 23;29(10):117557. doi: 10.1016/j.isci.2026.117557 (PMC13634412; doi:10.1016/j.isci.2026.117557)
Supplement: Supplementary file 1 [file mmc1.pdf]

iScience, Volume *29*

Supplemental information

MED26 facilitates Mediator-Pol II complex assembly and promotes nuclear stress body formation during heat stress

Kexin Li, Kazuki Furugori, Hidefumi Suzuki, Ryota Abe, Shun Sawatsubashi, Nanami Yasui, Yoko Ike, Keiko Horiuchi, and Hidehisa Takahashi

Supplemental Information, Li K, et al.

Figure S1 is related to Figure 1.

Figure S2 is related to Figure 2 and Figure 3.

Figure S3 is related to Figure 3.

Figure S4 is related to Figure 4 and Figure 5.

Figure S5 is related to Figure 6.

Figure S1

A

	NT-Score	HS-Score	HS/NT
MED31	17.24	19.27	1.117
MED27	14.50	15.89	1.096
MED9	90.82	96.27	1.060
MED17	84.06	86.73	1.032
MED15	61.08	58.29	0.954
MED10	112.39	104.27	0.928
MED28	27.95	25.60	0.916
MED8	57.45	51.94	0.904
MED13L	5.92	5.35	0.904
MED30	43.44	39.22	0.903
MED1	10.27	9.25	0.901
MED14	52.56	47.30	0.900
MED19	73.65	65.17	0.885
MED26	337.51	298.04	0.883
MED21	104.34	87.76	0.841
MED7	26.85	22.04	0.821
MED22	160.73	129.49	0.806
MED11	18.95	14.46	0.763
MED16	65.12	48.69	0.748
MED23	172.50	127.48	0.739
MED29	67.01	48.97	0.731
MED6	62.31	44.05	0.707
MED18	11.29	7.77	0.688
MED24	17.85	11.82	0.662
MED25	87.50	57.84	0.661
MED4	40.90	26.94	0.659
MED20	140.93	79.79	0.566
MED12	15.91	4.56	0.287

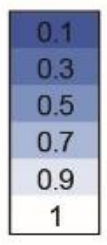

B

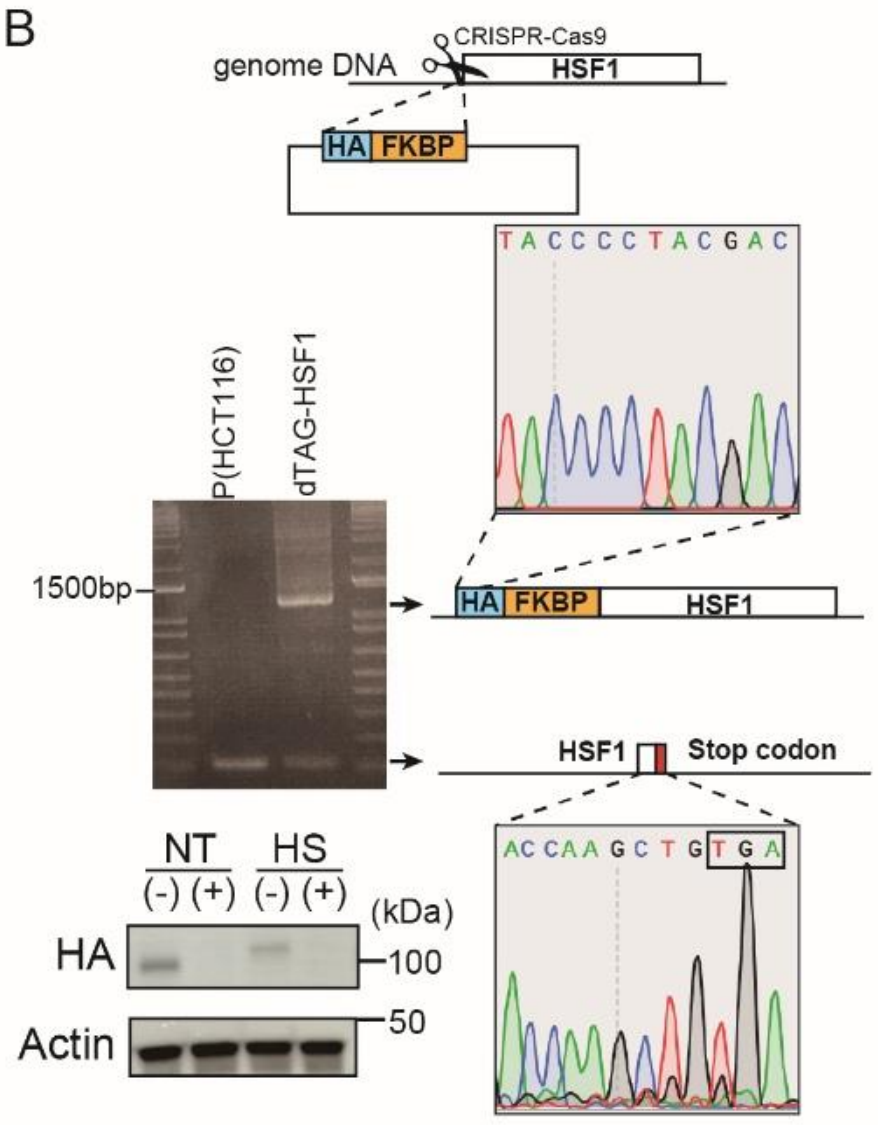

C

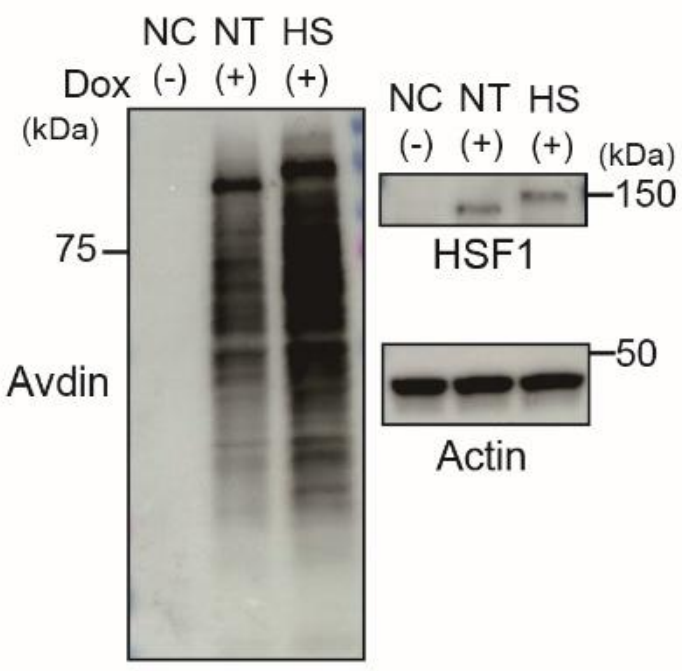

D

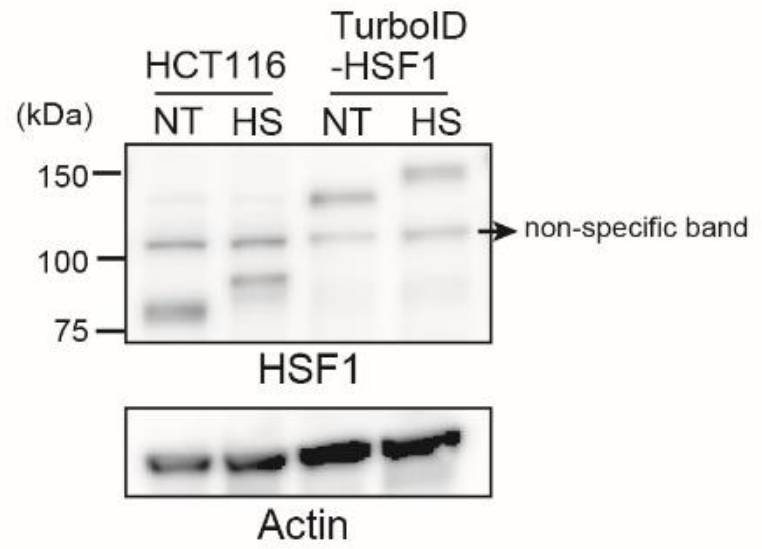

E

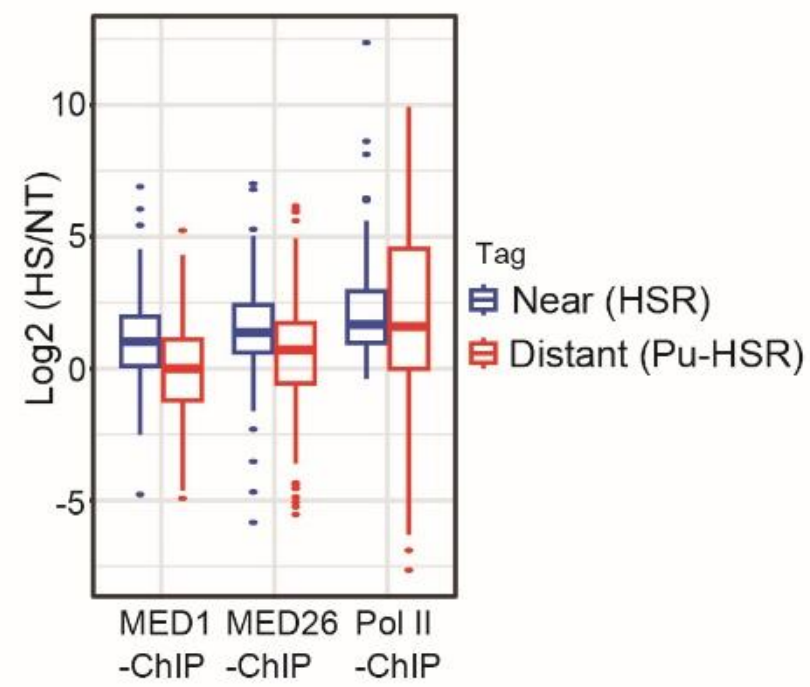

F

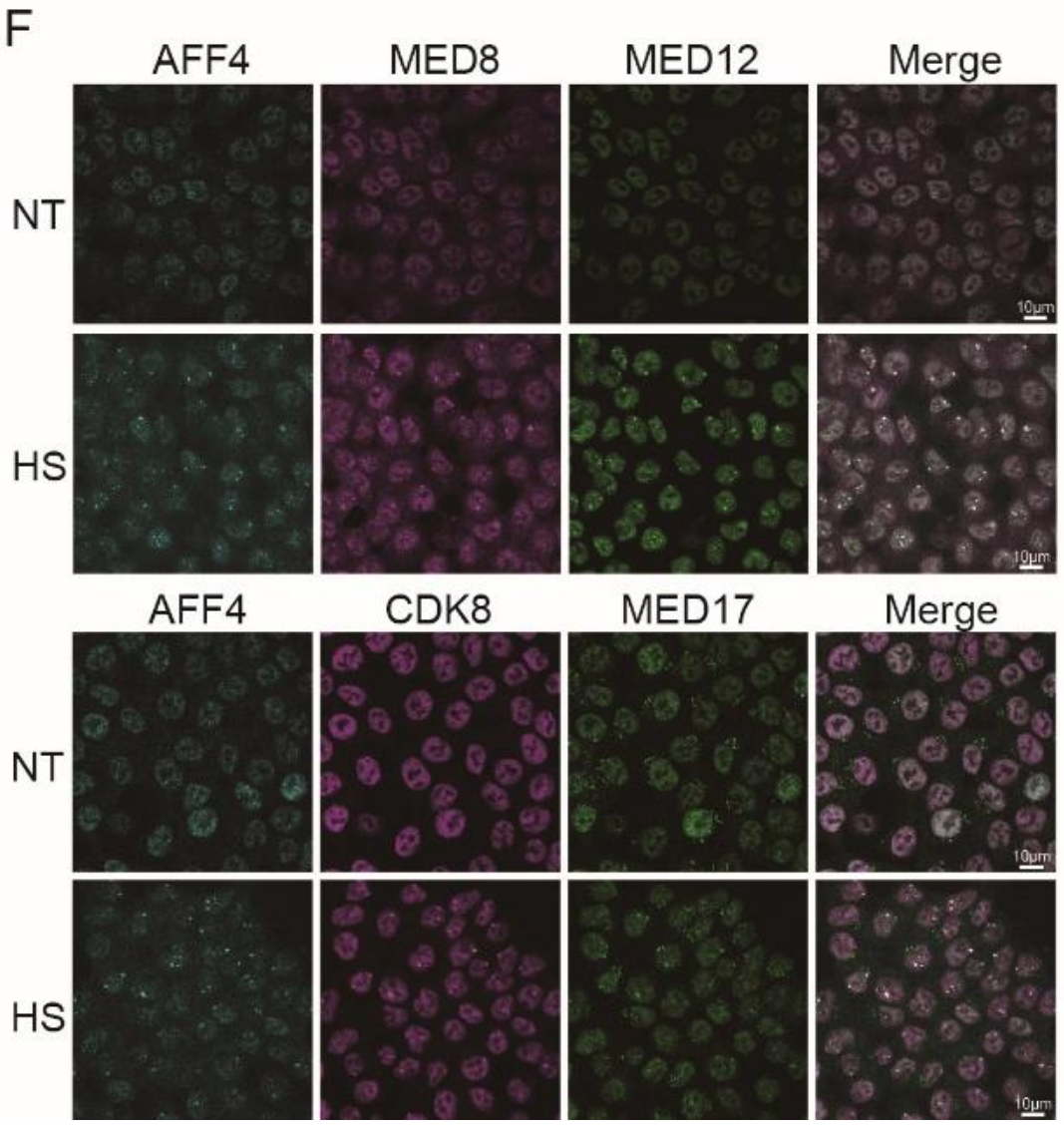

G

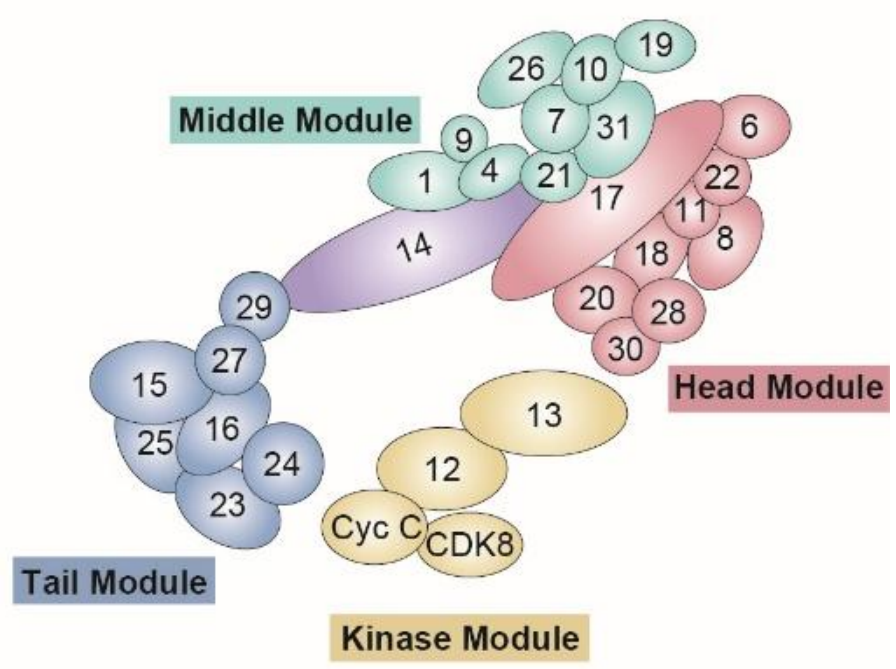

Figure S1: Heat stress induces the translocation of MED26-containing Mediator to nuclear stress bodies (nSBs) via interaction with HSF1. (A) Subunits of Mediator complex found to interact with MED26 under normal temperatures (NT) and heat stress (HS) in FLAG-MED26 cells, identified by MS. Proteins are listed in descending order, indicating decreased interaction of MED12 with MED26 under HS, and the proteins excluding MED12 were largely unchanged. The IP-MS experiment was independently repeated three times. **(B)** Confirmation of dTAG-HSF1 cells. Upper panel: Schematic illustration of the HSF1-depleted cells produced by CRISPR-Cas9. Middle panel: PCR-genotyping and Sanger sequencing of dTAG-HSF1 cells, and the results showing that one allele was successfully knocked in, while the other allele showed a frameshift causing a premature stop codon, leading to a functional knock out. Lower panel: Confirmation of HSF1 knockdown after dTAG treatment by western blotting. The western blotting experiment was independently repeated three times with similar results, and a representative blot is shown. **(C)** Expression of HSF1 in cells stably expressing TurboID-HSF1 and incubated with 100 μM biotin for 1 h to biotinylate proteins in close proximity to HSF1 upon simultaneous heat shock (HS; 43°C) or a normal temperature (NT). Dox was added at 300 ng/mL. NC, negative control. The experiment was independently repeated three times with similar results, and a representative blot is shown. **(D)** Comparison of HSF1 expression in TurboID-HSF1 cells and the parent cell HCT116 under HS or NT. The experiment was independently repeated three times with similar results, and a representative blot is shown. **(E)** Box plots showing the log2 fold changes in MED1, MED26, and Pol II ChIP-seq signals following heat shock treatment relative to the non-treated condition (HS/NT). A total of 271 genes significantly upregulated upon heat shock treatment (adjusted p value < 0.05, log2 fold change > 2, and baseMean > 10) were included in the analysis. Among these genes, those with an HSF1 peak located within 5 kb of the transcription start site (TSS) were classified as HSR genes, whereas the remaining genes, whose TSSs were located more than 5 kb from the nearest HSF1 peak, were classified as putative HSR (Pu-HSR) genes. Each data point represents an individual gene. n represents two independent biological replicates for each ChIP-seq experiment ($n = 2$). **(F)** IF images of the Mediator complex and SEC components, showing colocalization of MED8 (magenta) and MED12 (green) with AFF4 (cyan) (upper panel) and colocalization of CDK8 (magenta) and MED17 (green) with AFF4 (cyan) (lower panel) in nSBs following HS treatment. Scale bars: 10 μm. The imaging experiment was independently repeated three times with similar results, and representative images are shown. **(G)** Schematic illustration of Mediator complex containing Head, Middle, Tail, and Kinase modules.

Figure S2

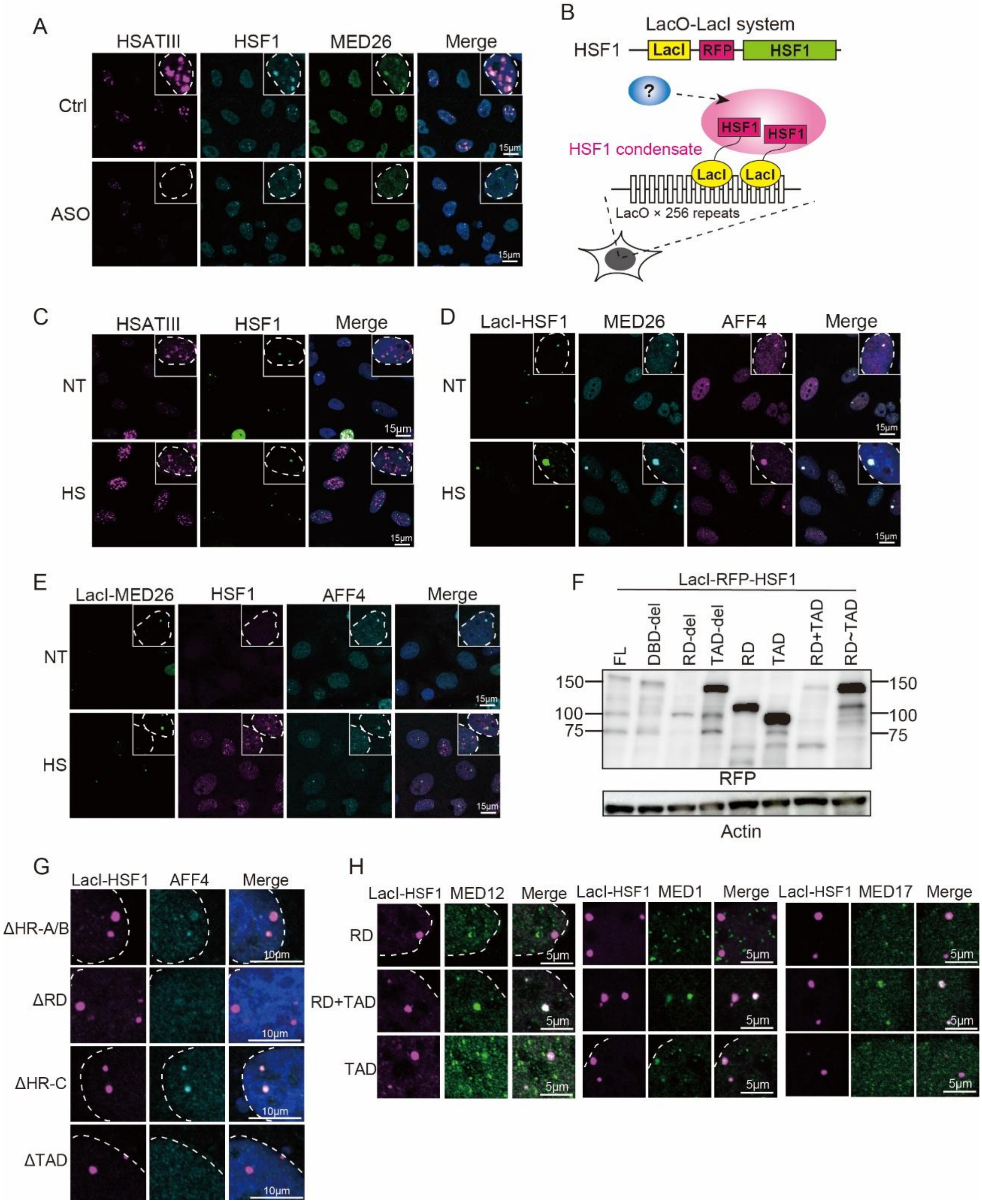

Figure S2: Interaction between HSF1 and MED26-containing Mediator depends on the RD and TAD of HSF1. (A) RNA-FISH images of HeLa cells following ASO-mediated knockdown of HSATIII lncRNA under heat shock (HS) conditions. Compared with the no-knockdown control (Ctrl), the size of HSF1 particles (cyan) decreased modestly under knockdown, while MED26 (green) retained colocalization with the small HSF1 condensates. Scale bars = 15 μm. The inset

shows an independently acquired representative image. **(B)** Schematic of the LacO-LacI system used to construct artificial HSF1 condensates for the identification of recruited proteins in U2OS cells. **(C)** RNA-FISH images of the LacO-LacI system, showing no colocalization of HSATIII lncRNA with LacI-HSF1 foci. Instead of LacI-RFP-HSF1, LacI-GFP-HSF1 was used in panels C and D. Scale bars: 15 µm. The inset shows an independently acquired representative image. **(D)** IF images of LacI-HSF1 (green), MED26 (cyan), and AFF4 (magenta), showing recruitment of intrinsic MED26 and AFF4 to the LacI-HSF1 foci. Instead of LacI-RFP-HSF1, LacI-GFP-HSF1 was used in panels C and D. Scale bars: 15 µm. The inset for the NT condition shows an independently acquired representative image, whereas the inset for the HS condition shows a magnified view of the corresponding low-magnification image. **(E)** IF images of LacI-MED26 (green), showing that intrinsic HSF1 (magenta) was not recruited to MED26 artificial condensates, while intrinsic AFF4 (cyan) was recruited to LacI-MED26 foci. Scale bars: 15 µm. The inset shows an independently acquired representative image. **(F)** Expression of LacI-RFP-HSF1-mutants. FL: Full length (~150–160 kDa); DBD-del: DBD deletion (~140–150 kDa); RD-del: RD deletion (~120–130 kDa, lower than expected, likely due to the loss of multiple PTM sites in RD); TAD-del: TAD deletion (~136–146 kDa); RD (~110–120 kDa); TAD (~90–100 kDa); RD+TAD (~130–140 kDa); RD~TAD: RD, HR-C, and TAD (~130–140 kDa). The experiment was independently repeated three times with similar results, and a representative blot is shown. **(G)** IF images of LacI-RFP-HSF1-mutants (magenta) after 1 h of HS. ΔRD, regulatory domain (IDR) deletion; ΔTAD, transactivation domain (IDR) deletion. Scale bars: 10 µm. Deletion of RD, TAD, or HR-A/B decreased colocalization of the SEC subunit AFF4 with HSF1. **(H)** IF images of lacI-RFP-HSF1 (magenta) and Mediator subunit (green) using only RD, TAD, or RD plus TAD HSF1. Scale bars: 5 µm. Recruitment of Mediator subunits, including MED1, MED12, and MED17, to HSF1 requires both the RD and the transactivation domain (TAD) of HSF1. The imaging experiments in (A), (C–E), (G), and (H) were independently repeated three times with similar results, and representative images are shown.

Figure S3

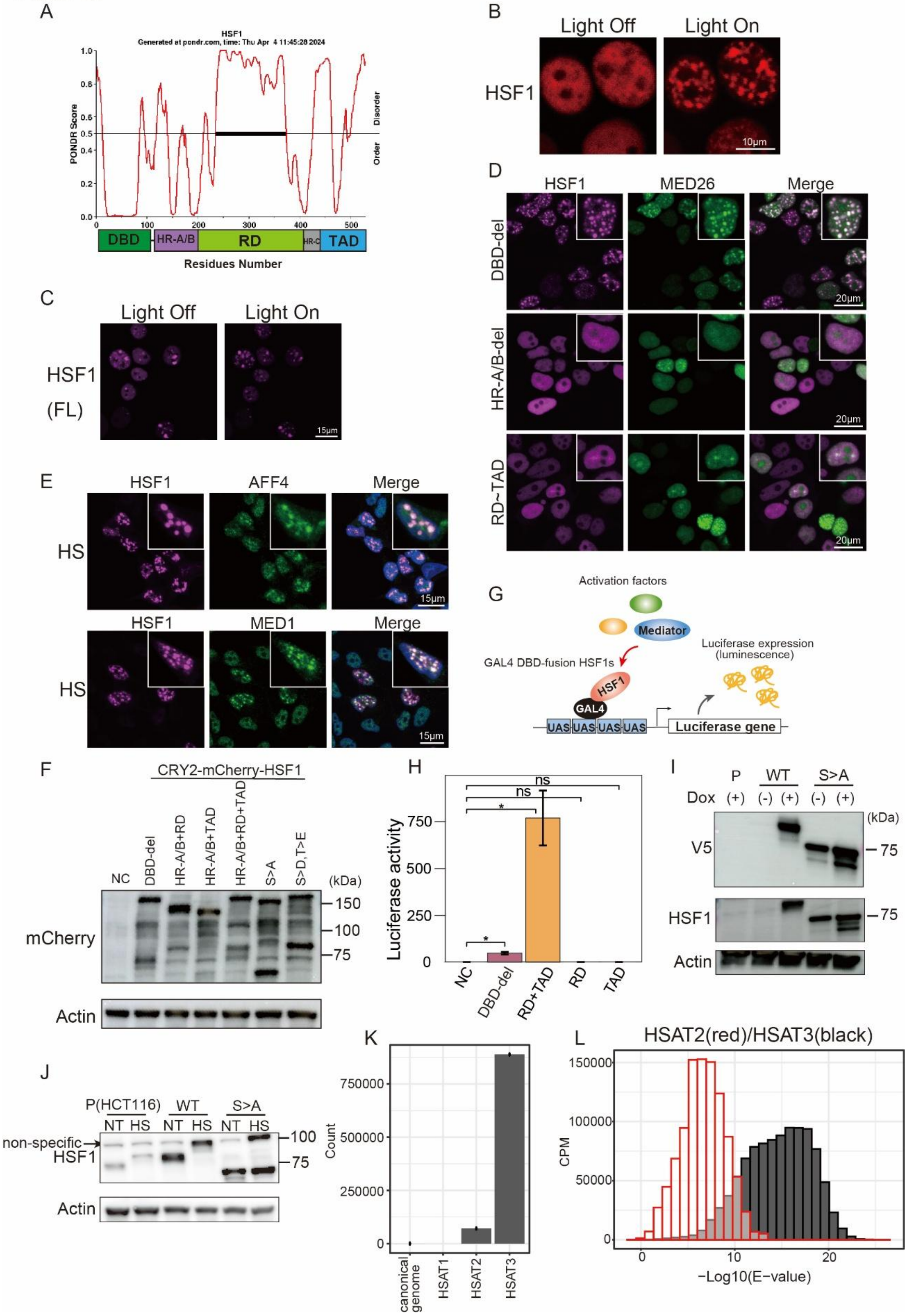
A
HSF1
Generated at pondr.com, time: Thu Apr 4 11:45:28 2024
PONDR Score
Disorder
Order
DBD
HR-A/B
RD
HR-C
TAD
Residues Number
B
Light Off
Light On
HSF1
10μm
C
Light Off
Light On
HSF1
(FL)
15μm
D
HSF1
MED26
Merge
DBD-del
HR-A/B-del
RD~TAD
20μm
E
HSF1
AFF4
Merge
HS
HSF1
MED1
Merge
HS
15μm
G
Activation factors
Mediator
GAL4 DBD-fusion HSF1s
HSF1
GAL4
UAS UAS UAS UAS
Luciferase gene
Luciferase expression
(luminescence)
F
CRY2-mCherry-HSF1
NC
DBD-del
HR-A/B+RD
HR-A/B+TAD
HR-A/B+RD+TAD
S>A
S>D,T>E
(kDa)
mCherry
150
100
75
Actin
H
Luciferase activity
ns
ns
*
*
NC
DBD-del
RD+TAD
RD
TAD
I
P
WT
S>A
Dox
(+)
(-)
(+)
(-)
(+)
(kDa)
V5
75
HSF1
75
Actin
J
P(HCT116)
WT
S>A
NT
HS
NT
HS
NT
HS
non-specific
HSF1
100
75
Actin
K
Count
canonical genome
HSAT1
HSAT2
HSAT3
L
HSAT2(red)/HSAT3(black)
CPM
−Log10(E−value)

Figure S3: Heat shock-induced hyperphosphorylation of the RD–TAD region is essential for HSF1 droplet formation and subsequent recruitment of MED26-containing Mediator. (A) IDR prediction of HSF1 using Predictor of Natural Disordered Regions (PONDR). The disordered region is indicated by the black horizontal line, with domains shown below. **(B)** Live cell images of the DBD-deleted HSF1 HSF1-mCherry-CRY2 fusion protein under normal temperature (NT) conditions, with and without light, in HEK293T cells. Scale bars: 10 µm. **(C)** Live cell images of full-length (FL)-HSF1-CRY2 under NT, showing the formation of HSF1 condensates with and without light, indicating binding to HSEs. Scale bars: 15 µm. **(D)** IF images using the indicated HSF1-CRY2 mutant fusion proteins (magenta) under HS conditions and light exposure. Scale bars: 20 µm. The inset shows a magnified view of the corresponding low-magnification image. **(E)** IF images of DBD-deleted HSF1-CRY2 (magenta), AFF4 (green), and MED1 (green) under HS conditions and light exposure. Scale bars: 15 µm. The inset shows an independently acquired representative image. The imaging experiments in (B–E) were independently repeated three times with similar results, and representative images are shown. **(F)** Expression of CRY2-mCherry-HSF1 mutants. DBD-del (~160–170 kDa); HR-A/B+RD (~130–140 kDa); HR-A/B+TAD (~120–130 kDa); HR-A/B+RD+TAD (~160–170 kDa); S>A (~150–160 kDa); S>D, T>E (~160–170 kDa). The experiment was independently repeated three times with similar results, and a representative blot is shown. **(G)** Schematic of the Luciferase assays used to confirm the transcriptional activity of HSF1 mutants. The GAL4-DBD (DNA-binding domain) fused to HSF1 mutants binds the UAS sequence, and the recruitment of activation factors such as Mediator complex by HSF1 mutants activates luciferase gene expression. **(H)** Fluorescence intensity of luciferase reporter assays of HSF1 mutants under normal temperature (NT) conditions, showing minimal transactivation by HSF1 containing only the RD or TAD. ns, not significant. *$P < 0.05$, calculated by pairwise two-tailed Student's *t*-tests in R. Data represent three independent experiments (n = 3), each performed in triplicate. **(I)** Western blotting validation of the stable expression of HSF1 mutants in HCT116 cells. P, parent cells with knockout of endogenous *HSF1*; WT, wild type; S>A, serine residues in the HSF1 IDR substituted with alanine. HSF1 expression was induced by Dox in WT cells, and HSF1 was expressed in S>A-expressing cells without Dox. **(J)** Comparison of HSF1 expression in WT-HSF1 and S>A-HSF1 stably expressing cells and the parent cell HCT116 upon HS or NT. The experiment in (I) and (J) was independently repeated three times with similar results, and a representative blot is shown. **(K)** Bar plot showing the detection of HSAT3 based on our RNA-seq data, and comparisons with the canonical genome, HSAT1 and HSAT2, showing that the HSAT3 detection sensitivity was approximately 80%. The analysis was based on three independent biological replicates of RNA-seq data (n = 3). **(L)** Bioinformatic analysis of HSAT2 and HSAT3 based on our RNA-seq data. The cutoff was set at 10. The analysis was based on three independent biological replicates of RNA-seq data (n = 3).

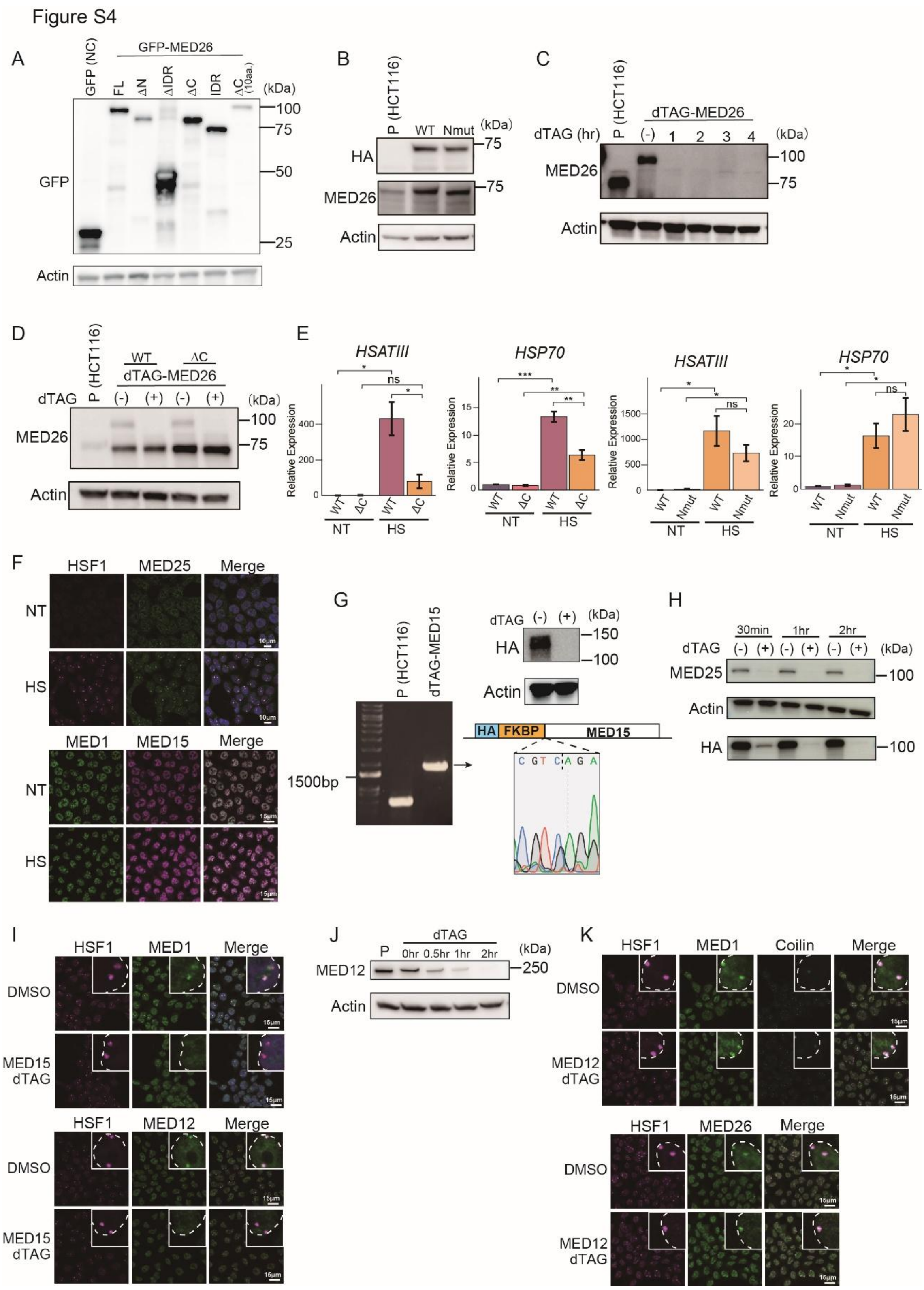

Figure S4: MED15 is responsible for MED26-Mediator colocalization with nSBs. (A)

Expression of EGFP-MED26 mutants. FL: Full length; ΔN: NTD-deletion; ΔIDR: IDR-deletion; ΔC: CTD-deletion; ΔC (10 aa): deletion of the 10 amino acids of MED26-CTD that interact with the Mediator complex. **(B)** Expression of MED26-WT and Nmut stably expressing cells. MED26-Nmut (R61A, K62A) showed no interaction with SEC. **(C)** Western blotting showing MED26 expression in dTAG-MED26 cells, MED26 was knocked down after adding dTAG for 1 h. **(D)** MED26-WT and MED26-ΔC stable expression in dTAG-MED26 cells. The western blotting experiments in (A–D) were independently repeated three times with similar results, and representative blots are shown. **(E)** qRT-PCR analysis of HSATIII lncRNA and *HSP70* expression in rescued cells expressing MED26-wild type (WT), ΔC, or N-mut. ns, not significant. $^{*}P < 0.05$; $^{**}P < 0.01$; $^{***}P < 0.001$, calculated using unpaired two-tailed Student's *t*-tests in R (n = 3 independent biological replicates). **(F)** IF images showing colocalization of MED15 and MED25 with MED1 and HSF1 (nSB) upon heat shock (HS). Upper panel: Antibodies against HSF1 and MED25 were used. Scale bars: 10 µm. Lower panel: Antibodies against MED1 and HA-tag (Human Influenza Hemagglutinin tag) were used to validate their localization in dTAG-MED15 cell lines. HA-tag was expressed with intrinsic MED15. MED1 colocalized with HSF1 in nSBs upon HS was validated in our earlier experiments. Scale bars: 15 µm. **(G)** Confirmation of dTAG-MED15 cells. Left panel: PCR-genotyping and Sanger sequencing of dTAG-MED15 cells. Right panel: Confirmation of MED15 knockdown after dTAG treatment by western blotting. The western blotting experiment was independently repeated three times with similar results, and a representative blot is shown. **(H)** Western blotting showing MED25 expression in dTAG-MED25 cells treated with dTAG for the indicated times. The dTAG-MED25 cell line was also validated by genotyping. The experiment was independently repeated three times with similar results, and a representative blot is shown. **(I)** IF images showing the near disappearance of MED1 and MED12 colocalized with HSF1 under MED15 knockdown. Scale bars: 15 µm. The inset shows an independently acquired representative image. **(J)** Western blot analyses showing that MED12 was knocked down after adding dTAG in dTAG-MED12 cells for 2h. The experiment was independently repeated three times with similar results, and a representative blot is shown. **(K)** IF images showing that the colocalization of MED1, MED26, and Coilin was not affected by MED12 knockdown. Scale bars: 15 µm. The inset shows an independently acquired representative image. The imaging experiments in (F), (I), and (K) were independently repeated three times with similar results, and representative images are shown.

Figure S5

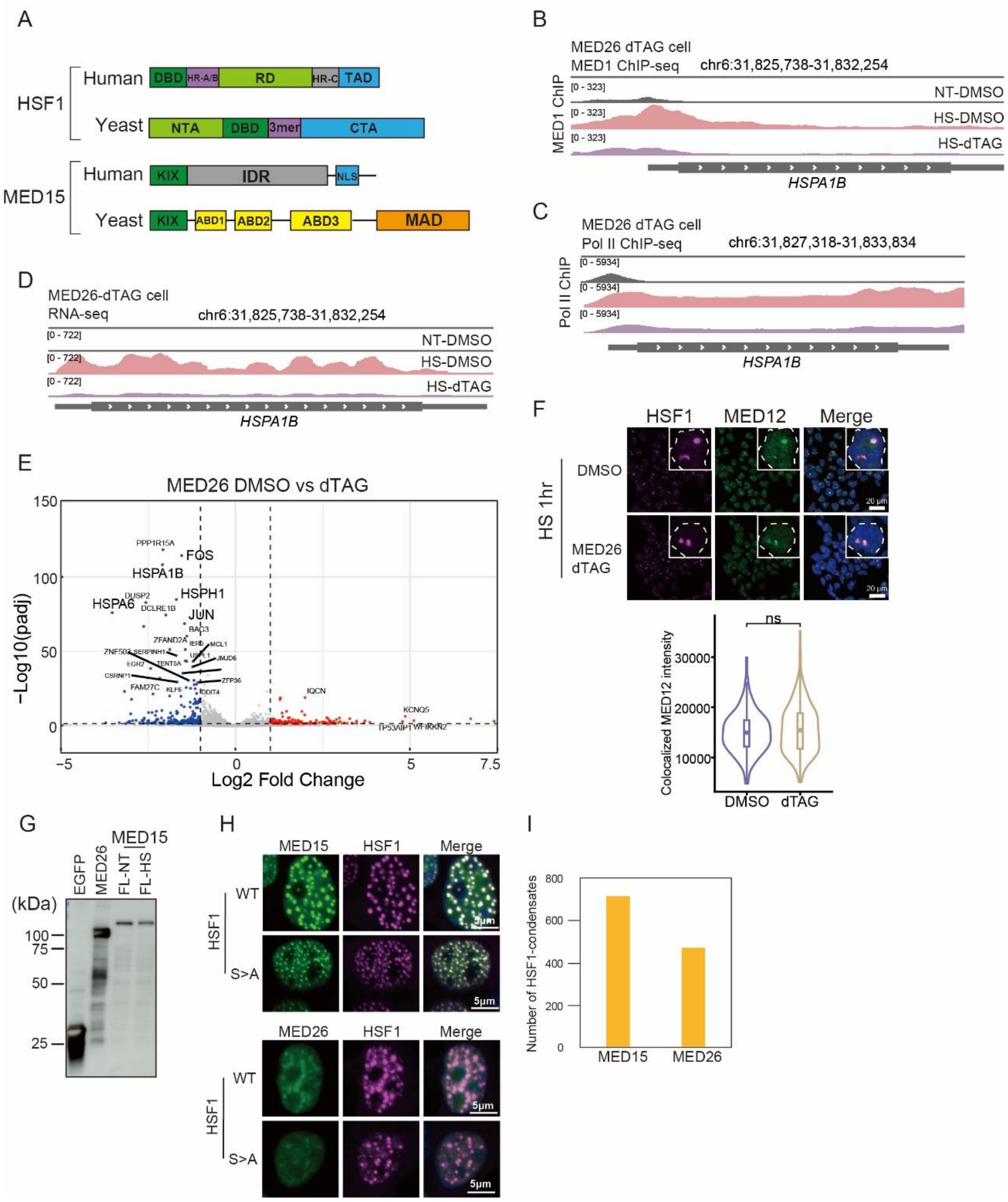

Figure S5: Deletion of the MED26 C-terminal region markedly reduces the expression of HSATIII lncRNA and *HSP70* genes. (A) Domain structure of HSF1 and MED15 in humans and yeast. KIX, SREBP1-binding domain; ABD, activator binding domain; MAD, mediator association domain. Human and yeast HSF1 share only 17% sequence identity and exhibit distinct domain organizations, with yeast Hsf1 containing an additional N-terminal TAD within its DBD

and lacking the HR-C region. **(B)** Wiggle tracks of the HSPA1B locus (chr6: 31825738–31832254) from MED1-ChIP-seq in dTAG-MED26 cells. A representative track from two independent biological replicates is shown; both replicates exhibited a consistent overall pattern. **(C)** Pol II-ChIP-seq wiggle tracks of the HSPA1B locus (chr6: 31827318–31833834) in dTAG-MED26 cells. A representative track from two independent biological replicates is shown; both replicates exhibited a consistent overall pattern. **(D)** RNA-seq wiggle tracks of the HSPA1B locus (chr6: 31825738–31832254) in dTAG-MED26 cells following acute depletion of endogenous MED26 by dTAG treatment. A representative track from three independent biological replicates is shown; all replicates exhibited a consistent overall pattern. **(E)** Volcano plot of RNA-seq of dTAG-MED26 cells, showing downregulation of many genes, particularly HSR genes, upon heat shock (HS) under MED26 knockdown. Genes with log2 fold change > 1 and $P < 0.05$ are highlighted in red, whereas genes with log2 fold change < −1 and $P < 0.05$ are highlighted in blue. n represents three independent biological replicates (n = 3). **(F)** IF images of HSF1 (magenta) and MED12 (green) in cells under MED26 knockdown and HS. Scale bars: 20 µm. The inset shows a magnified view of the corresponding low-magnification image. Right panel: violin-plot of MED12 fluorescent intensity colocalized with HSF1. ns, not significant. Each data point represents an individual MED12 focus (DMSO, n = 250 foci; dTAG, n = 270 foci). The experiment was independently repeated three times with similar results. **(G)** Western blotting validation of transiently expressed EGFP, EGFP-MED26, and EGFP-MED15. Notably, the expression level of MED15 is lower than that of MED26. The experiment was independently repeated three times with similar results, and a representative blot is shown. **(H)** IF images of CRY2-HSF1 (magenta), MED15 (green), and MED26 (green) under normal temperature conditions. Scale bars: 5 µm. The imaging experiment was independently repeated three times with similar results, and representative images are shown. **(I)** Number of CRY2-HSF1 condensates without heat stress, showing greater condensate formation for CRY2-HSF1 co-transfected with MED15 than for that with MED26 under normal temperature conditions. n represents the number of individual cells analyzed (n = 65 cells). Data were obtained from three independent experiments.
